# TH2BS11ph histone mark is enriched in the unsynapsed axes of the XY body and predominantly associates with H3K4me3-containing genomic regions in mammalian spermatocytes

**DOI:** 10.1186/s13072-019-0300-y

**Published:** 2019-09-07

**Authors:** Iyer Aditya Mahadevan, Satyakrishna Pentakota, Raktim Roy, Utsa Bhaduri, Manchanahalli R. Satyanarayana Rao

**Affiliations:** 1 Molecular Biology and Genetics Unit, Jawaharlal Nehru Centre for Advanced Scientific Research, Jakkur PO., Bangalore, 560064 India; 2 Novo Nordisk Foundation Center for Protein Research, Faculty of Health and Medical Sciences, University of Copenhagen, Blegdamsvej 3B, 2200 Copenhagen, Denmark; 3 The Graduate School of the Stowers Institute for Medical Research, 1000E. 50th St., Kansas City, MO 64110 USA

**Keywords:** TH2B serine 11 phosphorylation, Spermatocytes, TH2B, Immunofluorescence, Coimmunoprecipitation, H3K4me3 co-association

## Abstract

**Background:**

TH2B is a major histone variant that replaces about 80–85% of somatic H2B in mammalian spermatocytes and spermatids. The post-translational modifications (PTMs) on TH2B have been well characterised in spermatocytes and spermatids. However, the biological function(s) of these PTMs on TH2B have not been deciphered in great detail. In our attempt to decipher the unique function(s) of histone variant TH2B, we detected the modification in the N-terminal tail, Serine 11 phosphorylation on TH2B (TH2BS11ph) in spermatocytes.

**Results:**

The current study is aimed at understanding the function of the TH2BS11ph modification in the context of processes that occur during meiotic prophase I. Immunofluorescence studies with the highly specific antibodies revealed that TH2BS11ph histone mark is enriched in the unsynapsed axes of the sex body and is associated with XY body-associated proteins like Scp3, γH2AX, pATM, ATR, etc. Genome-wide occupancy studies as determined by ChIP sequencing experiments in P20 C57BL6 mouse testicular cells revealed that TH2BS11ph is enriched in X and Y chromosomes confirming the immunofluorescence staining pattern in the pachytene spermatocytes. Apart from the localisation of this modification in the XY body, TH2BS11ph is majorly associated with H3K4me3-containing genomic regions like gene promoters, etc. These data were also found to corroborate with the ChIP sequencing data of TH2BS11ph histone mark carried out in P12 C57BL6 mouse testicular cells, wherein we found the predominant localisation of this modification at H3K4me3-containing genomic regions. Mass spectrometry analysis of proteins that associate with TH2BS11ph-containing mononucleosomes revealed key proteins linked with the functions of XY body, pericentric heterochromatin and transcription.

**Conclusions:**

TH2BS11ph modification is densely localised in the unsynapsed axes of the XY body of the pachytene spermatocyte. By ChIP sequencing studies in mouse P12 and P20 testicular cells, we demonstrate that TH2BS11ph is predominantly associated with H3K4me3 positive genomic regions like gene promoters, etc. We propose that TH2BS11ph modification could act alone or in concert with other histone modifications to recruit the appropriate transcription or XY body recombination protein machinery at specific genomic loci.

## Background

Mammalian spermatogenesis offers an excellent model system to study chromatin remodelling by histone variants as the testis is known to express a large number of core and linker histone variants in a stage-specific manner [1–6]. It is hypothesised that chromatin locus-specific histone replacement with histone variants could be a possible basis for genome reprogramming in germ cells. Testis-specific histone variants play critical roles during the germ cell development. H3t (testis-specific histone variant of H3) is essential for the process of spermatogonial differentiation and ensures entry into meiosis [7]. The loss of TH2A and TH2B leads to male sterility with defects in cohesin release during interkinesis and histone to protamine replacement, suggesting an essential role of the testis-specific histone variants during critical periods of male germ development [8].

TH2B, a synonym of germ cell-specific H2B.1 (or TS H2B.1) [9] was one of the earliest histone variants discovered in mammalian testis [10, 11]. To date, it is the only testis-specific histone variant known to replace a core histone on a genome-wide scale replacing 80–85% of H2B in spermatocytes and spermatids [12]. The pachytene nucleosome core particle harbouring the TH2B molecule was shown to be less compact compared to H2B-containing nucleosome core particle [13, 14]. hTSH2B (human TH2B)-reconstituted histone octamer was found to be less stable than the H2B-reconstituted histone octamer in vitro [15]. On the other hand, the loss of mouse TH2B is compensated for upregulation of H2B and compensatory histone modifications on the core histones H2B, H3 and H4 in germ cells. However, the expression of tagged-TH2B protein created a dominant negative phenotype, resulting in a defective histone to protamine replacement that led to male sterility [12]. TH2B shares 85% sequence similarity with canonical histone H2B with majority of the differences being at its N-terminus. We surmised that these differences or the post-translational modifications acquired by some of the residues could contribute to unique functions of TH2B. In this direction, Pentakota et al. [16] characterised the repertoire of post-translational modifications (PTMs) on histone variant TH2B isolated from spermatocytes and spermatid stages. By computational analysis, it was also shown that the amino acid differences and the post-translational modifications acquired by some of the residues cause the destabilisation of TH2B-containing nucleosomes. Histone PTMs are key molecular players in epigenomic functions [17, 18]. Recently, various studies have focussed on understanding the post-translational modifications on testis-specific histone variants like TH2B [16, 19], TP1 (Transition Protein 1) [20], TP2 (Transition Protein 2) [20], HILS1 (Histone Linker H1 Spermatid specific 1) [21], etc.

During prophase I of meiosis, the homologous chromosomes synapse and undergo recombination at non-randomly selected loci. The exchange of genetic material is critical for the generation of diversity in the offspring. During leptotene interval, the global induction of Spo11-mediated DSBs occurs throughout the genome triggering the DNA damage response (DDR) [22, 23]. Subsequently, MRN (Mre11-Rad50-Nbs1) complex recruits ATM kinase, and catalyses the first level of H2AX phosphorylation to form γH2AX [24–26]. The end resection and strand invasion mediated by MRN complex, RAD51, DMC1 and other proteins are characteristic of the next stage, the zygotene interval. During the pachytene stage, BRCA1 senses asynapsis and recruits ATR kinase for amplification of DDR signals along the unsynapsed axes for the establishment of the γH2AX domain in the XY body [27–29]. The region of homology between the X and the Y chromosomes termed as pseudo-autosomal region (PAR) is limited in size (~ 800 kb in mice) and is largely unsynapsed during meiosis. Therefore, to ensure chromosome segregation with at least one crossover in PAR, a higher crossover density and increased DSB occurrence are observed in the PAR compared to that of autosomes [30]. The increase in DSB sites is caused by specialised chromatin configuration in PAR where DNA is organised on a longer axis with shorter chromatin loops compared to autosomes [31, 32]. The non-PAR regions of the X and Y chromosomes are transcriptionally silenced during meiotic prophase by a process termed as meiotic sex chromosome inactivation (MSCI) [33, 34]. The crossover products are generated at the end of pachytene [35]. The completion of meiosis I yields secondary spermatocytes which undergo meiotic II division to produce haploid round spermatids.

In our efforts to gain insight into the unique functions of TH2B particularly during meiotic prophase I, we detected a post-translational modification in the amino terminal end that was already reported in another study in round spermatid TH2B [36], Serine 11 phosphorylation on TH2B (TH2BS11ph according to Brno nomenclature for histone modifications [37]). Histone phosphorylation is linked to diverse biological processes like DNA damage and repair (DDR) [38], apoptosis [39], etc. In this study, we show that TH2BS11ph modification is a histone mark associated with unsynapsed axes of the XY body in pachytene spermatocytes of rodents. ChIP-sequencing studies further reveal that majority of TH2BS11ph-containing genomic regions were not hotspot-related but associated with other H3K4me3-containing regions like gene promoters, enhancers, etc (Table 1). Additionally, this histone mark is also associated with important proteins and histone marks linked to functions of gene regulation and XY body, as revealed by mass spectrometry studies. This is the first report documenting the role of a post-translational modification of a germ cell-specific core histone variant in meiotic prophase I-related events.

**Table 1:** List of datasets used for computational data analyses

Dataset	Geo accession IDs
DSB hotspots	GSE93955
TSS (of mouse)	GENCODE
H3K4me3	GSE35498
H3K4me3 common	GSE93955
H3K4me3 (B6 specific)	GSE93955
TH2B	GSE45915

## Results

### TH2B serine 11 phosphorylation (TH2BS11ph) is a novel histone modification detected in mammalian spermatocytes

**Fig. 1:**
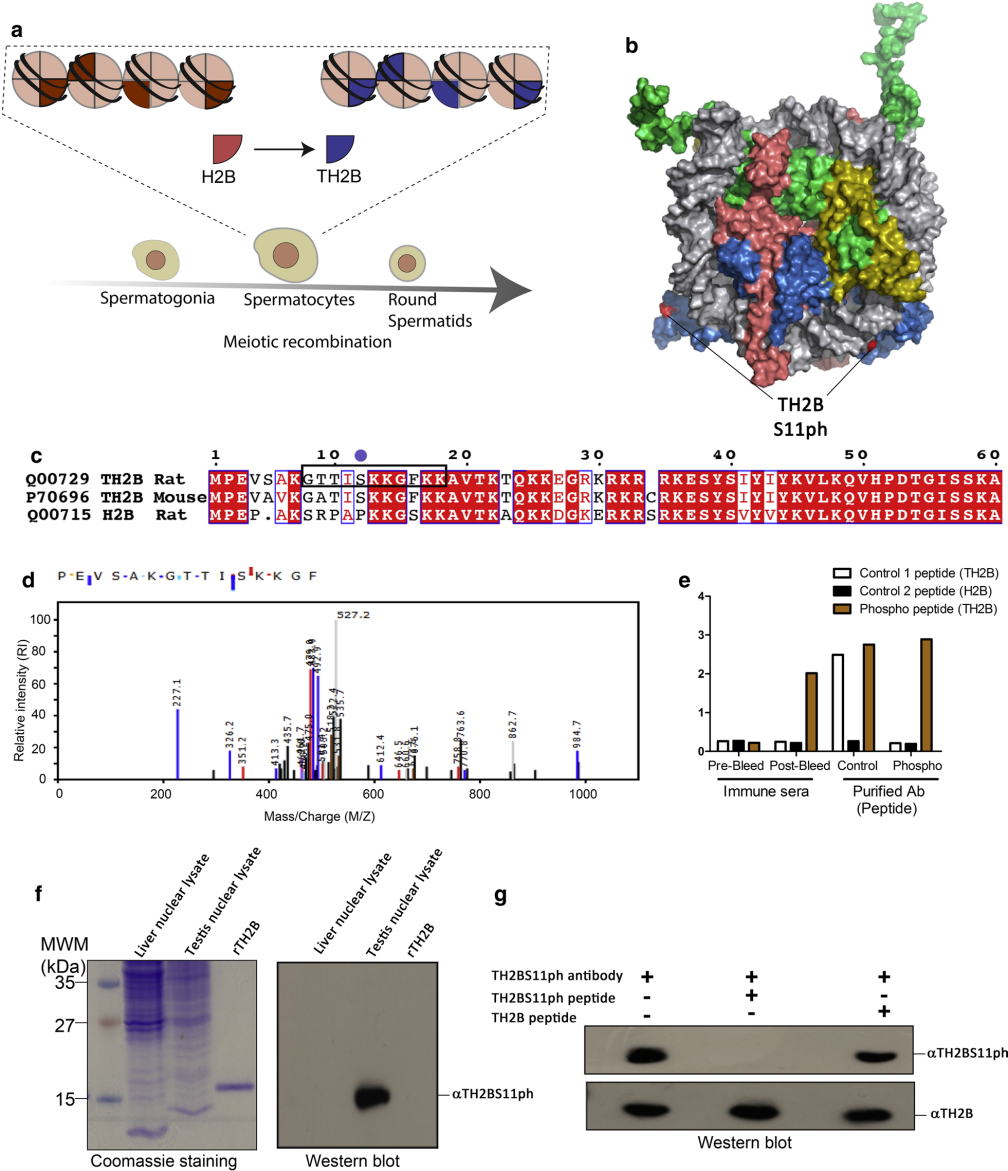
Identification of TH2BS11ph modification by mass spectrometry and characterization of the TH2BS11ph antibody. **a** Genome-wide replacement of H2B by TH2B histone variant in mammalian spermatocytes and spermatids [12]. **b** Model of the TH2B-containing nucleosome highlighting the exposed serine 11 residue. **c** Alignment of protein sequences of TH2B from rat and mouse. H2B sequence of rat has been given for reference. The boxed region in black represents the peptide sequence used for generation of the polyclonal antibody in rabbits. **d** Identification of TH2BS11ph modification by LC–MS/MS technique. The *y*-axis indicates the relative intensity of MS/MS spectra and the *x*-axis indicates the mass–charge ratio. The phosphorylated serine residue of TH2B-containing peptide fragment is highlighted in red. **e** ELISA assay—TH2B antibody reacts with both TH2B serine 11 phospho- and non-phosphopeptides, whereas the TH2BS11ph antibody specifically reacts with the TH2B serine 11 phosphopeptide. ELISA was carried out with the Pre-bleed immune sera, Immune sera, TH2BS11ph sera and the TH2BS11ph purified antibody; white bars represent reactivity of the mentioned sera or antibody with TH2B backbone peptide, black bars represent reactivity with H2B peptide and the orange bars represent reactivity with the TH2B serine 11 phosphopeptide. **f** Immunoblotting of affinity purified TH2BS11ph antibody shows reactivity with only TH2B-containing nuclear lysate. Western blotting was performed with anti-TH2BS11ph antibody against liver nuclear lysates, testes nuclear lysates and recombinant TH2B (rTH2B). Coomassie stained gel is given for reference on the left. **g** Peptide competition assay—first lane represents no peptide control, second lane TH2BS11ph antibody pre-incubated with TH2B serine 11 phosphopeptide, third lane TH2BS11ph antibody pre-incubated with TH2B backbone peptide. Western blot was carried out with the antibodies indicated as alpha alongside the blot

TH2B is the major histone variant in spermatocytes and spermatids [12] (Fig. 1a). As mentioned earlier, TH2B differs from H2B protein with majority of amino acid residues differing in the solvent exposed amino terminal tail (Fig. 1b, c). In an attempt to decipher various PTMs on TH2B, we purified in vivo TH2B from 30-day rat testicular cells by the reverse-phase HPLC technique. By employing a different set of procedures that includes enzyme digestion and post-mass spectrometry analyses of various PTMs obtained on in vivo TH2B, we detected the modification TH2BS11ph (TH2B serine 11 phosphorylation) in spermatocytes. This modification was already detected in TH2B from round spermatids by Luense et al. but not in spermatocytes [36]. We show for the first time the occurrence of this modification on TH2B isolated from spermatocytes. The representative MS/MS plot is shown in Fig. 1d. The corresponding fragmentation table has been given in Additional file 1: Fig. S1A. The somatic H2BS14ph modification has been shown to be involved in DNA repair processes in somatic cells [40]. Therefore, we were interested to ascertain the function of the testis-specific H2B variant TH2B serine 11 phosphorylation in the context of DNA repair and meiotic recombination associated events in spermatocytes. For this purpose, polyclonal antibodies specific to this modification were generated in rabbits.

The specificity of the antibody was determined by ELISA and western blot assays. ELISA assays were carried out with the TH2BS11ph antibody, wherein the antibody showed high reactivity towards the TH2B serine 11 phosphopeptide but not with the TH2B and H2B backbone peptides (Fig. 1e). The antibody showed reactivity towards testis nuclear lysates (Fig. 1f, Lane ‘Testis nuclear lysate’) but did not cross-react with the H2B containing liver nuclear lysate and also the recombinant TH2B protein by western blotting (Fig. 1f, lanes ‘Liver nuclear lysate’ and ‘rTH2B’). Further, the reactivity of the antibody was abolished by the TH2B serine 11 phosphopeptide (Fig. 1g, lane 2) but not by the TH2B unmodified peptide (Fig. 1g, lane 3). The combination of mass spectrometry and Western blotting analysis, thus, establishes that the TH2BS11ph modification is physiological.

### TH2BS11ph modification is densely localised in the axes of the XY body during the pachytene stage of meiosis prophase I

**Fig. 2:**
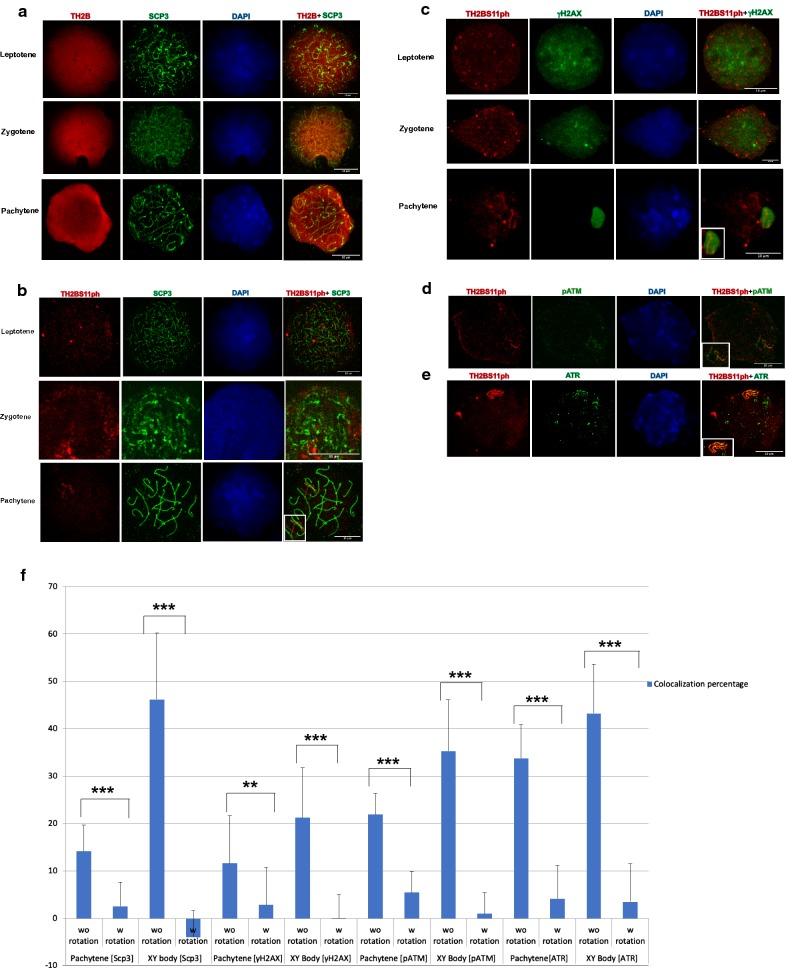
TH2BS11ph modification is densely localised in the axes of the XY body. **a** Immunofluorescence studies of backbone TH2B and Scp3 in leptotene (1st panel), zygotene (2nd panel) and pachytene stages (3rd panel) of meiotic prophase I. **b** Colocalization studies of TH2BS11ph modification with synaptonemal complex protein Scp3 across leptotene (1st panel), zygotene (2nd panel) and pachytene stages (3rd panel) of meiotic prophase I. The inset in the pachytene image represents the XY body. **c** Colocalization studies of TH2BS11ph with γH2AX in leptotene (1st panel), zygotene (2nd panel) and pachytene spermatocytes (3rd panel). The inset in the pachytene image shows the XY body. Immunofluorescence studies of TH2BS11ph with **d** pATM and **e** ATR kinases in pachytene spermatocytes. The insets in **d**, **e** show the XY body. Data information in (**a**–**e**); All data were confirmed with at least three independent mice (C57BL6 species). Nuclei were visualised by DAPI staining, Scale bars, 10 µm. **f** Quantitation of colocalization percentages of TH2BS11ph with Scp3, γH2AX, pATM and ATR in the whole pachytene spermatocyte and XY body. The colocalization percentages were calculated with (with rotation) and without (without rotation) image rotation. For calculation of colocalization percentages upon image rotation, the TH2BS11ph images captured in the red channel were rotated by 90° in the anticlockwise direction in the XY plane. The number of nuclei analysed are Scp3 (*n* = 10), γH2AX (*n* = 15), pATM (*n* = 15) and pATR (*n* = 15). The data are plotted in terms of mean ± SD ***P ≤ 0.0005, **P ≤ 0.005, *P ≤ 0.05 (*t*-test). *w rotation* with rotation, *wo rotation* without rotation

After establishing the high specificity of the TH2BS11ph antibody, we carried out immunofluorescence studies with the TH2BS11ph antibody to examine the staining pattern during meiotic prophase I. TH2B begins to express in preleptotene spermatocytes and continues to be present till the late stages of spermiogenesis [12]. Keeping this in mind, we carried out immunofluorescence staining of meiotic spreads of mouse testicular germ cells to examine whether the TH2BS11ph modification has any role in the events that are characteristic of meiotic prophase I. We have used Scp3 (Synaptonemal Complex Protein 3) and γH2AX as markers to distinguish various stages of meiotic prophase I in mouse [41] (Table 2). We observe that TH2BS11ph is detectable during the stages leptotene, zygotene and pachytene intervals as indicated in Fig. 2b. It is interesting to note that the backbone TH2B is detected all over the nucleus (Fig. 2a), while TH2BS11ph signal was more distributed as specific foci, suggesting a locus-dependent function. An important observation that is apparent in the staining pattern in pachytene spermatocytes is that TH2BS11ph modification was found to be highly enriched at the axes of the XY body-like structure (Fig. 2b, pachytene). This was further corroborated by colocalization analysis as represented in Fig. 2f (Scp3) which revealed that TH2BS11ph signal colocalizes with Scp3 in the XY body (Fig. 2f, pachytene without rotation and XY body without rotation). We found a higher colocalization percentage of TH2BS11ph and Scp3 of about 47% in the XY body compared to 14% in the whole pachytene spermatocyte (Fig. 2f pachytene without rotation, XY body without rotation). To evaluate the specificity of colocalization and to ensure that the observed signal is not a result of random overlap, we performed colocalization analyses after rotating images captured in the red channel by 90° in the anticlockwise direction. A significant decrease in colocalization percentages with usage of rotated images in comparison to non-rotated images would mean specific colocalization between the two channels. On rotation of the TH2BS11ph images captured in the red channel, we found that the colocalization percentage of TH2BS11ph and Scp3 decreased significantly in the XY body (Fig. 2f, Scp3, XY body with rotation) and in pachytene spermatocyte (Fig. 2f, Scp3, pachytene with rotation) in comparison with the non-rotated images. This indicates that TH2BS11ph is highly enriched in the axes of the XY body of the pachytene spermatocyte. We, therefore, conjectured that this modification may have a XY body-specific function in spermatocytes. This was also confirmed in meiotic spreads of rat testicular cells where we observed increased enrichment of TH2BS11ph signal in the XY body-like structure (Additional file 2: Fig. S2A, pachytene). Apart from the XY body of pachytene cells, we observed many foci outside the sex body suggesting the role of TH2BS11ph may not be just restricted to XY body-specific functions.

**Table 2:** List of antibodies used in the present study

Reagents				
Antibodies	Host	Company name	Cat number	Application
Scp3	Mouse	Abcam	ab97672	IF
γH2AX	Mouse	Upstate (Millipore)	05-636	IF, ChIP, WB
H2BS14ph	Rabbit	Scbt	sc31671	WB
pATM	Mouse	Upstate (Millipore)	05-740	IF
ATR	Mouse	Abcam	ab54793	IF
H3K4me3	Mouse	Abcam	ab12209	ChIP, WB
TH2B	Rabbit	Generated in house		IF, WB
Rad51	Mouse	Abcam	ab1837	IF
Spo11	Goat	Santa Cruz	sc22476	IF

*IF* immunofluorescence, *ChIP* chromatin immunoprecipitation, *WB* western blotting

H2AX is required for chromatin remodelling and sex chromosome inactivation in male meiosis [42]. We confirmed the enrichment and localization of this TH2B modification in the XY body of the pachytene nucleus using the sex body-specific marker γH2AX. As can be seen in Fig. 2c (pachytene), TH2BS11ph colocalizes with γH2AX corresponding to the axes of the XY body in the pachytene spermatocytes. The degree of colocalization of TH2BS11ph with γH2AX in the XY body was found to be 21% in the XY body as opposed to colocalization in the whole pachytene spermatocyte (~ 11%) as indicated in Fig. 2f (γH2AX, Pachytene without rotation, XY body without rotation). It is to be noted that γH2AX has a different staining pattern where it stains the axes and loops of the XY body, whereas TH2BS11ph stains only the axes; this might be the reason for lower colocalization percentages for γH2AX in the XY body. On rotation of TH2BS11ph images captured in the red channel, we found colocalization percentages to decrease significantly as shown in Fig. 2f (γH2AX, pachytene with rotation and XY body with rotation) in comparison with the non-rotated images. On the basis of colocalization observed with Scp3 and γH2AX in the axes of the sex body, we conclude that TH2BS11ph is densely localised to the axes of the XY body.

In a previous study, H2BS14ph was shown to stain the XY body of pachytene spermatocytes in mouse [40]. Since, our data also showed that TH2BS11ph localizes to XY axes, we wondered whether the TH2BS11ph antibody did crossreact with H2B or its posttranslational modifications in spermatocytes. However, on re-examination of the published data, we found that the same H2BS14ph commercial antibody cross-reacts also with in vivo TH2B (Additional file 1: Fig. S1C, in vivo TH2B). The antibody has been withdrawn and no more available. Therefore, we generated a H2B-specific antibody, validated its reactivity by dot-blot assay and western blotting with liver and testicular histones as shown in Additional file 1: Fig. S1D. We carried out staging of the H2B antibody with Scp3 to obtain the staining pattern at various stages of meiotic prophase I. The staining of backbone H2B was found to be not intense contrary to that previously reported for H2BS14ph modification in all the stages of meiotic prophase I (Additional file 1: Fig. S1E). Furthermore, H2B staining did not coincide with the XY body of the pachytene spermatocyte (Additional file 1: Fig. S1E, pachytene). This also supports the report wherein H2B levels were found to decrease completely at 18 dpp mouse testes that coincide with onset of differentiation of pachytene cells [12]. Nevertheless, the combination of Western Blotting with peptide competition, and ELISA assays prove that the TH2BS11ph antibody did not cross-react with H2B, further negating the previous report where H2BS14ph was shown to localise to the XY body suggesting that the reported staining pattern was a result of cross-reactivity with TH2B.

### TH2BS11ph modification is enriched in the unsynapsed axes and associated with recombination-related kinases like pATM and ATR in the XY body of the pachytene spermatocyte

From mid-zygotene interval, unsynapsed chromosomes are marked by ATR, where the latter carries out the second level of H2AX phosphorylation [27, 29]. pATM and ATR are the markers of the unsynapsed axes of the XY body [43]. To test the enrichment of TH2BS11ph in the axes of the XY body, we next sought to perform colocalization studies with pATM and ATR kinases. We observe colocalization with pATM in axes of the XY body as can be seen in Fig. 2d. Upon further quantitative analyses, we observe colocalization with pATM of about 35% corresponding to the axes of the XY body as indicated in Fig. 2f (pATM, XY body without rotation). On rotation of the TH2BS11ph images captured in the red channel, the colocalization percentage dipped to about 1%, suggesting the specific overlap of TH2BS11ph and pATM in the axes of the XY body in the non-rotated images (Fig. 2f, pATM, XY body with rotation).

To determine the association of XY body-specific kinase ATR and TH2BS11ph, colocalization studies were also carried out of TH2BS11ph and ATR. There was a distinct colocalization seen specifically in the axes of the XY body as seen in Fig. 2e. We also quantified the colocalization percentages between TH2BS11ph and ATR, where we found a high colocalization percentage of about 43% in the XY body (Fig. 2f, ATR, XY body without rotation). We found colocalization percentage of 33.8% in the whole pachytene spermatocyte (Fig. 2f, ATR, pachytene without rotation). The colocalization percentages decreased significantly on rotation of the TH2BS11ph images captured in the red channel (Fig. 2f, ATR, pachytene with rotation, XY body with rotation). On the basis of colocalization of TH2BS11ph with ATR and pATM in the XY axes during the pachytene interval, we conclude that this modification is densely localised to the unsynapsed axes of the XY body.

XY body harbours various proteins related to recombination and heterochromatin formation. DDR proteins that accumulate in the XY body, have different and distinct localisation patterns. Proteins like γH2AX are spread over the entire sex chromosomes (axes + loops), whereas ATR, pATM, Rad51 are localised in the unsynapsed axes [43]. This suggests that unsynapsed axes are different from other chromosomal regions of the sex body in terms of distinct localisation patterns of various DDR related proteins. Since pATM and ATR are marker proteins of the unsynapsed axes and colocalize with TH2BS11ph [43], we conclude that TH2BS11ph histone mark is enriched in the unsynapsed axes of the XY body of pachytene spermatocyte. Taken together, the immunofluorescence studies reveal that the TH2BS11ph modification is associated with the proteins γH2AX, pATM and ATR that are related to DNA recombination and repair processes in the XY body. Some of the key observations of the immunofluorescence data in mouse meiotic spreads were also shown to be true for rat testicular cells as shown in the Additional file 2: Fig. S2.

### TH2BS11ph modification is predominantly associated with H3K4me3-containing genomic regions in spermatocytes

**Fig. 3:**
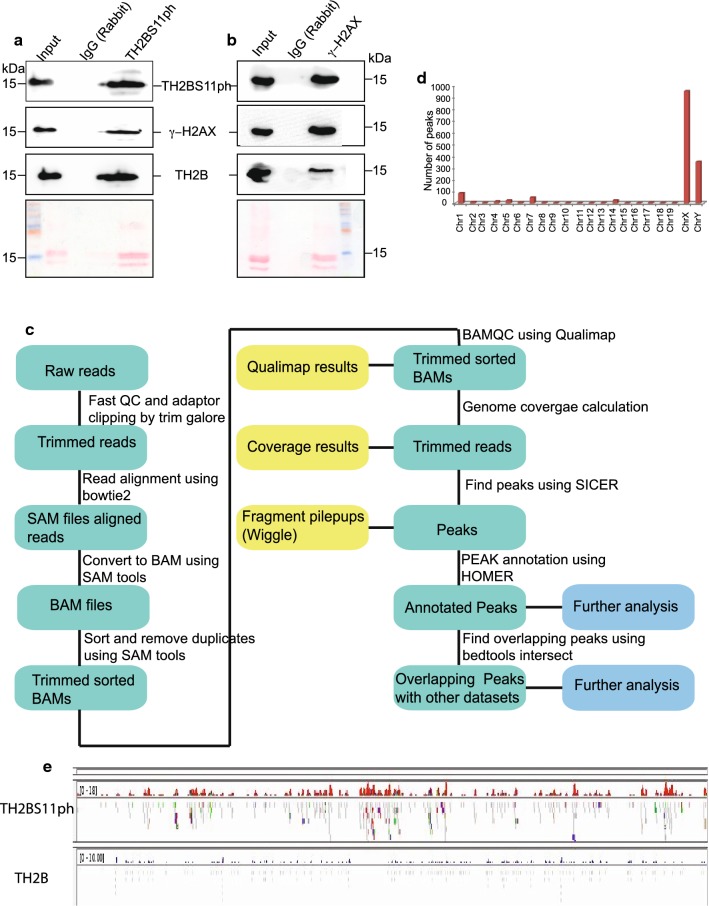
TH2BS11ph histone mark is predominantly associated with genomic regions containing the histone mark H3K4me3. TH2BS11ph-containing mononucleosomes associate with sex body-specific histone mark γH2AX-Mononucleosome IP studies determining the coexistence of histone marks TH2BS11ph with γH2AX, **a** γH2AX is associated with TH2BS11ph-containing mononucleosomes. IP was carried out using the anti-TH2BS11ph antibody where the TH2BS11ph, γH2AX and TH2B were probed by western blotting. **b** TH2BS11ph is associated with γH2AX-containing mononucleosomes. IP was carried out with the γH2AX antibody, and scored for TH2BS11ph, γH2AX, and TH2B by western blotting. The antibodies used for western blotting are indicated to the side of the blot. Ponceau stained blots have been given for reference. Data information in **a**, **b** The numbers represent molecular weight in kilodaltons. The first lane in all the blots represents the input fraction, 2nd lane IgG elute fraction and 3rd lane immunoprecipitation with the mentioned antibodies. **c** Workflow of ChIP sequencing analysis for TH2BS11ph histone mark carried out in mouse P20 testicular cells. **d** Chromosome wise distribution of TH2BS11ph peaks across chromosomes of mouse-majority of the TH2BS11ph peaks were found in chromosomes X and Y. **e** Distribution of TH2BS11ph and TH2B reads across the X-chromosome. For both TH2BS11ph and TH2B sections, the upper track represents the peaks, whereas the bottom track represents the read distributions as observed in IGV Genome Browser. TH2B ChIP sequencing data were taken from Gene Expression Omnibus (GSE45915) [12]

ATR kinase, not pATM, has been shown to be involved in XY body-specific phosphorylation of H2AX [29]. Therefore, we went ahead to examine whether TH2BS11ph is associated with the known sex body-specific marker, γH2AX. To verify the immunofluorescence studies further, we, therefore, carried out immunoprecipitation reactions with mononucleosomes to determine the coexistence of sex body-associated histone mark γH2AX in a single nucleosome core particle. We employed MNase digestion followed by immunoprecipitation to address whether TH2BS11ph is associated with γH2AX in the context of mononucleosomes. The profile of MNase digestion with respect to time of digestion has been given in Additional file 5: Fig. S5A. By IP assays, we demonstrate that TH2BS11ph pulled down γH2AX protein (Fig. 3a, TH2BS11ph). Conversely, TH2BS11P is also found in γH2AX elute fraction (Fig. 3b, γH2AX). These experiments suggest that TH2BS11ph and γH2AX co-associate possibly to recruit similar set of proteins in turn to regulate DSB processes in the XY body.

Since meiotic recombination is a non-random event and occurs at specific loci termed as meiotic recombination hotspots, we were curious to examine the possible presence of TH2BS11ph at these hotspot loci. Since the genetics of homologous recombination and hotspot genomic regions are well characterised in C57BL6 mouse species, and also many of the colocalization experiments were reproduced in both rat and mouse testicular cells, we were interested to determine the genome-wide occupancy of this modification in pachytene cells (P20 testicular cells) and in leptotene (P12 testicular cells). Immunofluorescence studies revealed the predominant localisation of TH2BS11ph in the unsynapsed axes of the XY body, also the localisation of this modification revealed many foci outside the XY body. Therefore, the localization of this modification in the context of binding sites on the pachytene genome had to be determined.

In this direction, ChIP-sequencing analyses of genome-wide distribution of TH2BS11ph was carried out to obtain a comprehensive picture of the association of TH2BS11ph with genomic regions in P20 mouse testicular cells enriched in pachytene cells. The workflow of the ChIP sequencing protocol and computational analyses is given in Fig. 3c. We used the already published TH2B ChIP-seq data for comparison that was carried out in elutriation-purified spermatocyte populations that usually is representative of TH2B occupancy in pachytene cells [12]. The important question was to determine the unique regions that are enriched for TH2BS11ph compared to backbone TH2B. To address this, we performed peak calling of TH2BS11ph ChIP seq data against the published TH2B dataset to obtain the unique peaks of TH2BS11ph occupancy. We observed high number of peaks in chromosomes X and Y (Fig. 3d, chrX and chrY). As can be seen in Fig. 3e, we also observe higher number of reads of TH2BS11ph IP in the X-chromosome in comparison to the backbone TH2B occupancy. This validated our immunofluorescence studies, wherein TH2BS11ph modification was found to be enriched in the XY body.

To identify the characteristic features of the genomic regions bound by TH2BS11ph histone mark, we were interested to check their overlap with meiotic recombination and transcription-related histone H3K4me3 ChIP-seq datasets [44]. To address this, we generated aggregation plots to determine the localisation of TH2BS11ph reads with respect to the centre of binding sites of total H3K4me3 marks, DSB hotspots and transcription-associated H3K4me3 mark [common H3K4me3], etc. All the analyses of overlap of TH2BS11ph with particular histone mark ChIP-seq datasets have been carried out in comparison with TH2B control dataset. We have generated two kinds of data to determine the overlap of TH2BS11ph with TSS or recombination hotspot-specific histone marks. Aggregation plot shows the concentration of read counts in respective genomic coordinates, i.e., TSS, etc. Heatmap are also generated for the corresponding aggregation plot to address the spatial distribution of reads within the proximity of the region of interest.

**Fig. 4:**
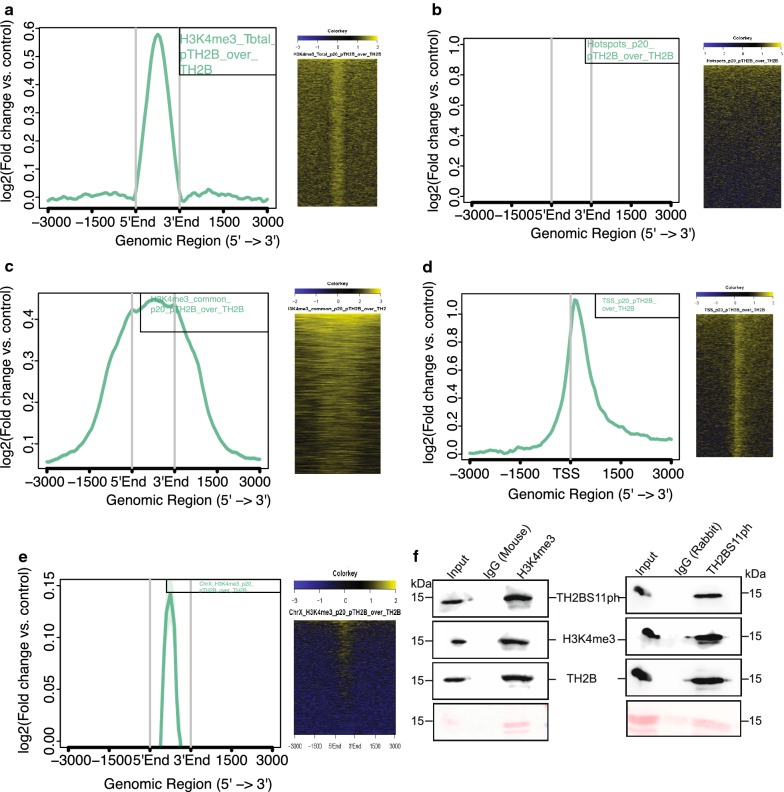
Localisation of TH2BS11ph at TSS and recombination hotspots with respect to TH2B. **a** Analysis of overlap of TH2BS11ph with Total H3K4me3. **b** Analysis of overlap of TH2BS11ph with DSB hotspots. **c** Overlap of TH2BS11ph with H3K4me3 common peaks representing the TSS-associated H3K4me3 marks. **d** Localisation of TH2BS11ph at TSS. **e** Overlap of TH2BS11ph with Chromosome X-associated H3K4me3 marks. The aggregation plots in **a**–**e** show the read coverage of unique TH2BS11ph reads (compared against TH2B) at ± 3 kb from the centre of the particular histone or protein marks as indicated in the boxes in the top right corner. *Y*-axis represents log2(fold change of TH2BS11ph versus TH2B control), *X*-axis represents distance in terms of kilobases. The heat map in **a**–**e** is the corresponding representation of the read coverage at ± 3 kb from the centre of occupancy of candidate histone/protein marks. **f** Association of H3K4me3 and TH2BS11ph in mononucleosomes. Mononucleosome IP studies to determine the coexistence of TSS-related histone mark H3K4me3 with TH2BS11ph histone modification by forward (1st panel) and reverse IP reactions (2nd panel). Ponceau stained blots have been given for reference. The first lane in all the blots represents the Input, second lane immunoprecipitation with the Mouse IgG antibody, third lane refers to the IP using the specific antibody (anti-H3K4me3 or anti-TH2BS11ph). The antibodies labelled alongside the blot refer to the antibodies that were used for western blotting

Aggregation plot as given in Fig. 4a demonstrates that majority of the reads were concentrated within 1 kb from the centre of occupancy of H3K4me3 mark (Fig. 4a, first panel). We also find secondary peaks further away from the centre of H3K4me3 mark, which might be due to the interaction of promoters with distal regulatory elements like enhancers, etc. The heat map representation corroborates with the fact the majority of TH2BS11ph reads lie at the centre of H3K4me3 peaks (Fig. 4a, second panel). The important thing to be kept in mind is that TSS and recombination hotspots also have similar histone mark profile consisting of H3K4me3, H3K36me3 marks, etc. Therefore, we were interested to delineate whether TH2BS11ph is linked to either TSS-specific or hotspot-specific histone mark H3K4me3. With respect to DSB hotspots, represented by occupancy of hotspot-specific H3K4me3 marks genome wide, we did not find significant overlap of TH2BS11ph at recombination hotspots as shown by aggregation plot given in Fig. 4b, (first panel). There were no reads concentrated at the centre of the DSB hotspots (Fig. 4b, first and second panels). We further looked at the association of TH2BS11ph with common H3K4me3 peaks representing the H3K4me3 marks present at gene regulatory regions like promoters, enhancers, etc. On further analysis, we found close association of TH2BS11ph with TSS (Transcription Start Sites) containing-H3K4me3 (Fig. 4c). Aggregation plot supports the conclusion wherein majority of the reads were concentrated within the centre of TSS-specific H3K4me3 (Fig. 4c, first and second panels). As the distance from H3K4me3 increases, the overlap of both these two histone marks also decreases. Therefore, the overlap of TH2BS11ph was specific to TSS-associated H3K4me3 but not hotspot-specific H3K4me3. We also generated Aggregation plot and heat map using available dataset of TSS of mouse. As can be seen in Fig. 4d, we observe specific localisation of TH2BS11ph reads at the centre of the transcription start sites further supporting the fact the specific association of this histone mark towards TSS (Fig. 4d, first and second panels). The presence of H3K4me3 is linked to high DSB activity in the PAR of the X-chromosome [45]. Furthermore, since we had found out that majority of TH2BS11ph peaks were in the X-chromosome, it became important to check the association with X-chromosome-specific H3K4me3 marks. We observed that TH2BS11ph-containing genomic regions were indeed positive for H3K4me3 mark in the X-chromosome (Fig. 4e, first and second panels). We have also plotted the read distribution of TH2BS11ph occupancy with respect to these datasets in Additional file 3: Fig. S3, wherein we observed the maximum overlap of TH2BS11ph reads at the centre of TSS and TSS-specific H3K4me3 marks (Additional file 3: Fig. S3C, D).

To determine the localisation and dynamics of localisation of the TH2BS11ph modification, we also performed ChIP-sequencing studies in mouse P12 testicular cells representative of leptotene/zygotene cells. Since various datasets for H3K4me3 and DSB hotspots are available for P12 mouse testicular cells, we wanted to compare the localisation and overlap of TH2BS11ph with these datasets in leptotene/zygotene cells. Aggregation plot for P12 TH2BS11ph ChIP sequencing data showed the predominant localisation with respect to total H3K4me3 marks (Additional file 4: Fig. S4A). Furthermore, the localisation was specific to transcription-specific H3K4me3 (common H3K4me3) but not recombination hotspot-specific H3K4me3 [B6 specific H3K4me3] as can be seen in Additional file 4: Fig. S4B, C, respectively. Even in leptotene cells, we observed specific association of TH2BS11ph towards TSS-associated H3K4me3 marks (Additional file 4: Fig. S4C). The defining factor of TH2BS11ph occupancy in leptotene and pachytene cells, therefore, also demonstrates association with TSS-associated H3K4me3 nucleosomes.

Open chromatin regions like transcription start sites are initiation for meiotic recombination in yeast [46]. However, hotspots and TSS are distinctly located in the mouse genome [47]. Aggregation plots and heat maps had showed strong association of TH2BS11ph with histone mark H3K4me3. To verify the ChIP sequencing results further, we were interested in carrying out immunoprecipitation reactions with mononucleosomes to determine the coexistence of histone mark H3K4me3 in a single nucleosome core particle. Therefore, we performed IP assays with mononucleosomes to prove the association of TH2BS11ph and H3K4me3 in the context of mononucleosomes. This interaction was also proved by IP assays, where co-IP with H3K4me3 antibody pulled down TH2BS11ph protein (Fig. 4f, H3K4me3 IP). Reciprocal IP also proves the association of H3K4me3 with TH2BS11ph-containing mononucleosomes (Fig. 4f, TH2BS11ph IP). Thus, we conclude that H3K4me3 is associated with TH2BS11ph-containing mononucleosomes.

### TH2B is depleted from active TSS and present at background levels at recombination hotspots in mouse spermatocytes

**Fig. 5:**
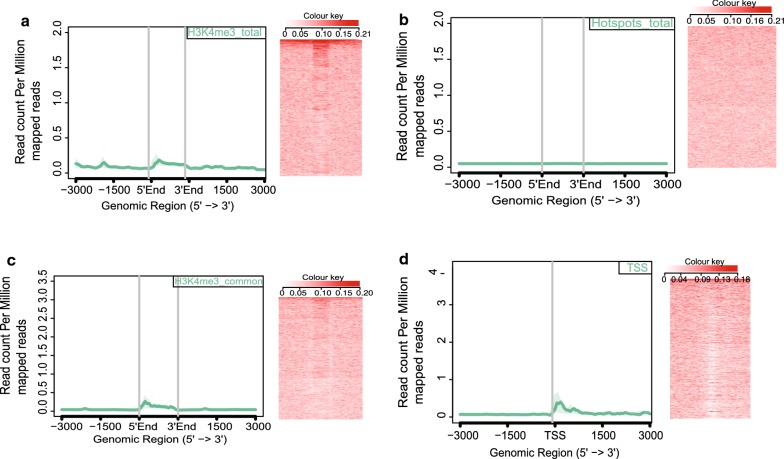
Localisation of backbone TH2B at TSS and recombination hotspots. Analysis of overlap between backbone TH2B with **a** total H3K4me3, **b** DSB hotspots, **c** H3K4me3 common representing the TSS-specific H3K4me3 marks. TH2B does not display preferential localization towards H3K4me3 (total), DSB hotspots and TSS-associated H3K4me3 histone marks. **d** Analysis of overlap of backbone TH2B with TSS of mouse. TH2B was observed to be depleted at TSS regions [12]. In each figure from **a**–**d**; the overlap has been determined using Aggregation plots and heat maps

TH2B replaces H2B on a genome-wide scale in spermatocytes and spermatids. Further, TH2B was previously reported to be depleted from transcriptionally active H2AZ-containing nucleosomes [12]. Since we have extensively proven the association of TH2BS11ph in the XY body and with TSS-associated H3K4me3, it became important to determine the level of backbone TH2B at those characteristic genomic loci like TSS and recombination hotspots. We observed that TH2B was present at background levels with respect to total H3K4me3 marks (Fig. 5a, first and second panels). We did not observe any specific enrichment of TH2B at recombination hotspots (Fig. 5b, first and second panels). Furthermore, the occupancy of TH2B reads was plotted against H3K4me3 common dataset, representing the TSS-related H3K4me3 marks; we did not find significant enrichment of TH2B in the proximity of TSS-specific H3K4me3 (Fig. 5c, first and second panels). We, therefore, reconfirmed the published dataset wherein we observe the depletion of TH2B at TSS regions; there was no preferential enrichment observed for TH2B at centre of known TSS genomic regions (Fig. 5d, first panel). Heat map also showed a characteristic depletion of TH2B reads at TSS regions (Fig. 5d, second panel). The important point to be noted is the read count per million mapped reads were plotted on the same scale as the observed TH2BS11ph levels at TSS and recombination hotspots. Taken together, we observe the specific enrichment of TH2BS11ph over TH2B at the H3K4me3-associated TSS regions. These analyses show that TH2BS11ph is preferentially enriched over TH2B in the XY body and H3K4me3-containing TSS regions.

### Validation of the ChIP sequencing dataset by ChIP-PCR

**Fig. 6:**
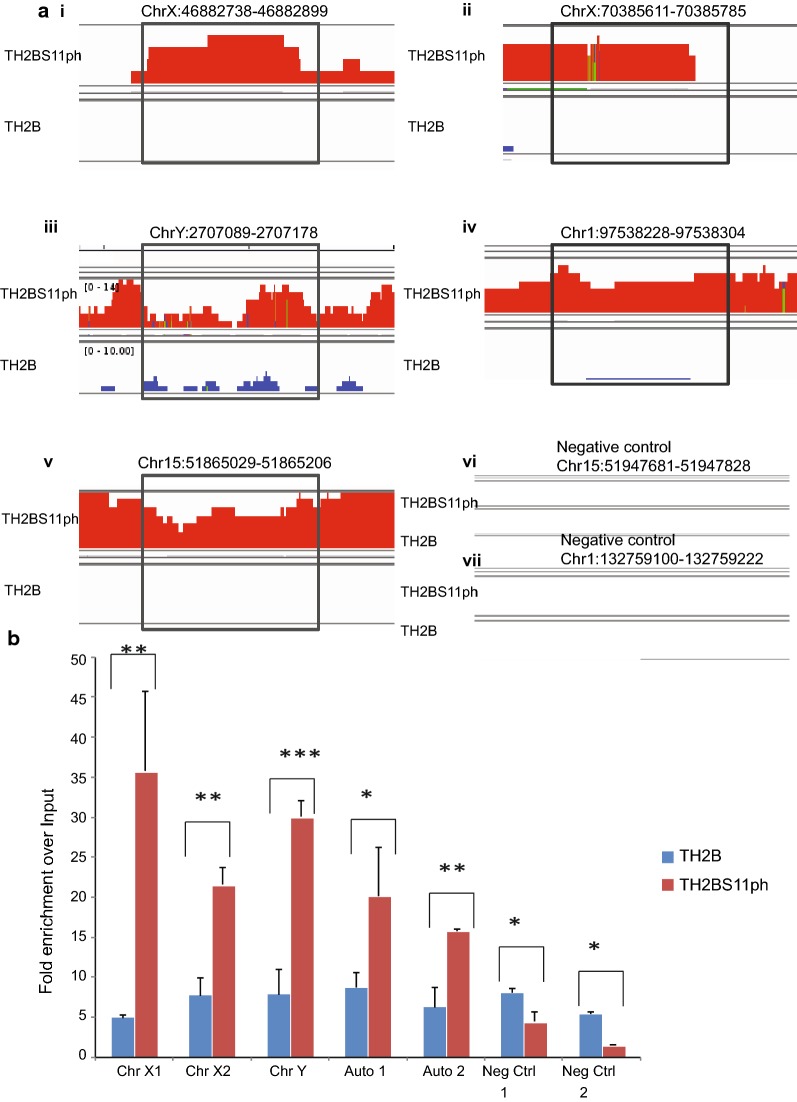
Validation of the ChIP sequencing dataset of TH2BS11ph in mouse P20 testicular cells. **a** Genomic regions used for designing primers required to confirm the ChIP-sequencing dataset by ChIP-PCR studies. Every top lane in all the panels from (i)–(vii) is TH2BS11ph IP, bottom panel-TH2B IP. **b** Validation of TH2BS11ph genome-wide occupancy data using ChIP-PCR technique using TH2B and TH2BS11ph antibodies. ChIP-PCR shows the enrichment of TH2BS11ph histone mark at the selected genomic regions associated with Chromosome X (2 regions), Chromosome Y and autosomes (2 regions) over TH2B IP and input control (i–v). A specific region in chromosome 15 and chromosome 1 were used as negative controls for this study (vi and vii). The fold enrichment values of TH2B and TH2BS11ph IPs were plotted against input control. ChIP-PCR experiments were done for three biological replicates including technical duplicates for a single biological replicate. The data are plotted in terms of mean ± SD, ***P ≤ 0.0005, **P ≤ 0.005, *P ≤ 0.05 (*t*-test)

For confirmation of the ChIP-sequencing dataset at selected loci, we designed primers for various chromosomal loci across the mouse genome. The genomic regions were determined for TH2BS11ph occupancy as can be in Fig. 6, in comparison to occupancy of backbone TH2B. The genomic regions with the TH2BS11ph and TH2B IP tracks that have been selected for experimental validation are shown in Fig. 6a. We have chosen two peaks from Chromosome X, one from Chromosome Y and two from autosomal regions for experimental validation (i–vii). By ChIP-PCR in P20 mouse testicular cells, we show the occupancy of TH2BS11ph at all the selected genomic regions, further validating the ChIP-seq results (Fig. 6b, ChrX1, ChrX2, ChrY, Auto1 and Auto2). Our main aim was to determine unique peaks of TH2BS11ph occupancy by comparing against the occupancy of backbone TH2B. TH2B ChIP was used as additional control to determine the occupancy of backbone TH2B at these genomic regions. We observe the specific enrichment of TH2BS11ph over TH2B as well as input control at all the selected genomic regions (Fig. 6b, ChrX1, ChrX2, ChrY, Auto1 and Auto2). There was no significant enrichment of TH2BS11ph over TH2B control in the selected regions of chromosomes 15 and 1 that were used as negative controls (Fig. 6b, Neg Ctrl 1 and Neg Ctrl 2).

### TH2BS11ph modification is associated with key proteins related to XY body and transcription

**Fig. 7:**
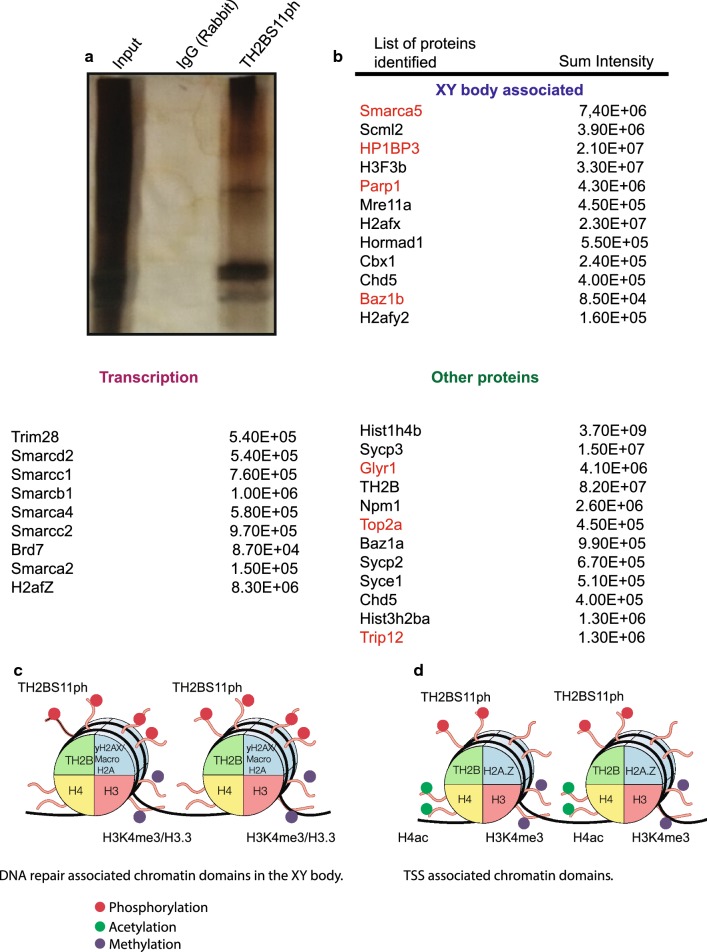
Determination of interacting protein partners of TH2BS11ph-containing mononucleosomes in rat testicular cells. **a** Silver-stained image of the TH2BS11ph ChIP-Input lane refers to 5% input; IgG lane refers to non-specific IgG control; TH2BS11ph refers to TH2BS11ph IP lane. **b** List of interacting proteins of TH2BS11ph-containing mononucleosomes as determined by mass spectrometry in rat testicular cells. The associated proteins are classified into three different categories based on their known functions: XY body, Transcription and other important proteins. The first row is the gene/protein name; the second row refers to the sum intensity that refers to the peak intensity values for all the peptides matched to a particular protein. The proteins highlighted in red refer to common proteins identified between TH2BS11ph IP and γH2AX IP (as reported by Broering et al. [48]). **c** Model of TH2BS11ph-containing nucleosomes showing the association with XY body-related histone marks. TH2BS11ph could function along with γH2AX and H3K4me3 histone marks to mediate DNA repair in the XY body. **d** Model of TH2BS11ph-containing nucleosomes showing the association of this histone mark with transcription start sites. TH2BS11P could associate with H3K4me3, H2AZ and transcription-associated H4 acetylated marks to mediate transcription in pachytene cells. TH2BS11ph could function in association with specific repertoire of histone marks to mediate chromatin-templated events like DNA repair in the XY body or TSS activation

Since TH2BS11ph is a nucleosomal histone protein, we were interested to determine the proteins that are associated with TH2BS11ph-containing mononucleosomes. To address this question, we performed mass spectrometry of the immunoprecipitated mononucleosomes using TH2BS11ph antibodies. We have again validated the specificity of the TH2BS11ph antibody, where we demonstrate that the TH2B Serine 11 phosphopeptide competes with the antibody in the immunoprecipitation (Additional file 5: Fig. S5B). The proteins were identified on the basis of enrichment of immunoprecipitated proteins with respect to those in non-specific pre-immune IgG lane (Fig. 7a). Mass spectrometric analyses revealed key proteins associated with the functions of XY body and transcription as shown in Fig. 7b further revalidating the ChIP-seq results where we observed the predominant association of TH2BS11ph with important genomic regions related to XY body and transcription.

Smarca5 is known to regulate Tyr142 phosphorylation on H2AX. It also associates with Cer2 to regulate gene expression. It was identified as the interacting protein partner of γH2AX [48]. Scml2, a germline specific subunit of Polycomb complex, is involved in epigenetic reprogramming of sex chromosomes [49]. Scml2 also promotes heterochromatin organisation in post-meiotic spermatids, by regulating the localization of CENP-V at pericentric heterochromatin [50]. Remodelling of the sex chromosomes is brought about by replacement of H3 by H3.3 wherein DSB proteins and chromatin remodelers act in sync to bring about MSCI induced nucleosome replacement [51]. Mre11 is a part of MRN complex associated with DSB events in the XY body [52]. Consistent with the localization of TH2BS11ph in the XY body, many proteins associated with the XY body functions like Scml2 [49, 50], Cbx1 [53], macroH2A [54], H2AX [42], H3.3 [51] were identified in the mass spectrometric analyses. Apart from this, chromosomal axes proteins like HORMAD1 that are involved in checkpoint surveillance required for meiotic progression [55] is also associated with TH2BS11ph mark. HORMAD1 preferentially is localised to unsynapsed chromosomes during the prophase I of meiosis [56]. It is also known that Cbx1/HP1β and macroH2A are co-associated with the PAR region of the XY body during male meiosis [57]. This reconfirms the association of TH2BS11ph with the XY body as both these proteins also shown to interact with TH2BS11ph-containing mononucleosomes.

H2AZ-containing nucleosomes are sites of transcription activation [58]. The fact that TH2BS11ph associates with proteins like SWI/SNF complex members, Trim28, H3.3, H2AZ, etc. establishes the fact that the TH2BS11ph-containing nucleosomes are also TSS associated. Synaptonemal complex proteins like Scp1, Scp2 and Scp3 were found to be associated with TH2BS11ph mononucleosomes [59–65]. Synaptonemal complex proteins are implicated in proper crossover formation and completion of meiosis [66]. Trim28 is known to associate with Brdt to mediate transcriptional repression in spermatogenic cells [67]. Npm1 is a known histone chaperone. In this list, we also obtained core nucleosomal histones, thus serving as internal control for the isolation of mononucleosomes.

In comparison to the proteins that were shown to be associated with γH2AX-containing nucleosomes in another report [48], we find that many proteins (highlighted in red) were common and also associated with TH2BS11ph-containing mononucleosomes (Fig. 7b). This suggests that both these histone marks associate with similar set of protein players in spermatocytes. The important point we would like to stress here is that TH2BS11ph histone mark also associates with proteins related to transcriptional regulation as shown by mass spectrometry. This indicates that TH2BS11ph associates with different set of protein machineries in turn regulating different loci in a context-dependent manner. Taken together, a recurrent theme that emerges from our study is that the histone mark TH2B Serine 11 phosphorylation is densely localised in the unsynapsed axes of the XY body, predominantly associate with H3K4me3-containing genomic regions and also associate with protein players involved in processes of XY body and transcription-related processes.

## Discussion

TH2B, a testis-specific histone variant, globally replaces somatic histone H2B in mammalian spermatocytes and spermatids [12]. Although, the biological function of TH2B has been delineated in spermatids in the context of histone to protamine transition, very little is known about its role in spermatocytes. The major differences between TH2B and its somatic counterpart H2B, is the sequence variation in the N-terminus tail. Histone tails do not exhibit specific structures in high-resolution crystal structures. However, they contribute to stabilisation of chromatin through contacts with DNA backbone and acidic patch of adjacent nucleosomes. Tail domains also provide the stabilisation effect by suppressing the accessibility of the DNA and regulating nucleosome mobility [68, 69]. The present study was initiated to explore the possible biological function(s) of the N-terminus of TH2B and associated histone modifications particularly with respect to the biological functions in spermatocytes. We have recently described the complete repertoire of TH2B PTMs from pachytene spermatocytes and round spermatids [16]. Our revisit to the mass spectrometric analysis of TH2B PTMs using a modified procedure of enzyme procedure and processing of peptides revealed Serine 11 phosphorylation on TH2B. This TH2BS11ph histone mark was reported by Luense et al. [36] only in round spermatids, but not in spermatocytes. This serine is highly conserved in TH2B of most of the mammals suggesting possible role(s) in germ cells. Our present study describes our detailed analysis of TH2BS11ph modification towards our understanding the biological function in the context of processes characteristic of meiotic prophase I in rodent spermatocytes.

### TH2BS11ph and the XY body

One of the major observations of the present study is the enrichment of TH2BS11ph in the axes of the XY body in pachytene spermatocytes as evidenced by both immunostaining pattern as well as ChIP-seq analysis. The key biological events that occur in the XY body are late DSB repair and heterochromatin formation. Based on the observation that TH2BS11ph is co-associated with γH2AX as demonstrated by immunolocalization as well as pull down experiments, we would like to believe that TH2BS11ph is associated with chromatin domains undergoing DSB repair in the XY body. This conclusion is also supported by the fact that somatic H2BS14ph has been shown to be associated with DNA repair chromatin domains in somatic cells [40]. The intense staining in the axes of the XY body could be due to increased DSB density that occurs in the PAR (Pseudo-Autosomal Region) of the XY body as compared to autosomal DSBs [31]. Importantly, a specialised chromatin configuration is found in the XY body wherein chromatin is organised into larger axes and shorter loops promoting higher DSB density. The fact that TH2BS11ph associated proteins also share similar protein interactome with γH2AX proteins [48] provides further support for our hypothesis that TH2BS11ph is associated with DNA repair domains within the XY body. It is also interesting to note that we also observed that several proteins associated with heterochromatinization were also present in the TH2BS11ph associated mononucleosomes like Scml2, macroH2A, HP1β, HP1γ, etc. Our studies also show the association of TH2BS11ph with X-chromosome-specific H3K4me3 marks. It is known that H3K4me3 catalysed in the PAR of the XY body is PRDM9 independent. This raises the question as to which kinase(s) is(are) responsible for the formation of TH2BS11ph-associated H3K4me3 mark. Taken together, these results strongly suggest that TH2BS11ph is an important histone mark associated with XY body-specific DSB repair and subsequent heterochromatinization. TH2BS11ph along with specific histone marks like H3K4me3, γH2AX, macroH2A could contribute to these specialised chromatin structure in the XY body, thus contributing to recombinational repair and heterochromatinization in the XY body (Fig. 7c).

### TH2BS11ph and transcription

Another major observation made in the present study is the association of TH2BS11ph with H3K4me3 mark. H3K4me3 has strong association with promoters, enhancers and meiotic recombination hotspots. The factors that determine selection of hotspots versus transcription start sites are the specific enzymes that catalyse the creation of H3K4me3 marks. PRDM9 catalyses H3K4me3 and H3K36me3 formation at recombination hotspots, whereas the enzyme(s) responsible for H3K4 trimethylation at transcription start sites in spermatocytes is(are) speculated to be SET/MLL enzymes [70].

Genomic-occupancy overlap studies using aggregation plots and heat maps obtained from ChIP-seq experiments in P20 testicular cells (pachytene) demonstrated the specific association of TH2BS11ph reads with TSS-specific H3K4me3 marks, but not the hotspot-related H3K4me3 marks. This was also proved by forward and reciprocal IP assays where we show the co-association of TH2BS11ph with H3K4me3 mark in vivo. This co-association with H3K4me3-positive TSS-associated genomic regions were also found to be true in P12 testicular cells (leptotene). It is quite likely that punctuate staining of TH2BS11ph observed outside the XY body in pachytene nuclei might correspond to transcriptional active chromatin domains. The association of TH2BS11ph with H3K4me3 marks within TSS is further demonstrated by the association of transcription-associated proteins like H2AZ, H3.3, SWI SNF complex members with the TH2BS11ph immunoprecipitated mononucleosomes as revealed by our mass spectrometric analysis. Taken together, we have demonstrated the strong association of TH2BS11ph with TSS-specific chromatin domains and histone mark H3K4me3 in vivo in pachytene spermatocytes.

As mentioned earlier, the N-terminal domain of H2B is involved in stabilisation and mobilisation of nucleosomes. In addition to the N-terminal tail, the HBR (Histone H2B Repression) domain in H2B (30–37 amino acid residues) is also involved in transcriptional repression. Acetylation of the lysine residues within HBR domain is shown to relieve the repression phenomenon and also facilitate transcription [71]. Our present study shows for the first time that phosphorylation of serine 11 in TH2B to be strongly associated with TSS mononucleosomes in pachytene spermatocytes. It remains to be seen whether serine 14 phosphorylation in somatic histone H2B is also associated with transcription in somatic cells. This is quite possible since the N-terminal is not structured and hence serine 11 phosphorylation in TH2B and serine 14 may have similar functions. Alternatively, the function of TH2B serine 11 phosphorylation may be unique to spermatocyte transcription.

In conclusion, we have demonstrated that TH2BS11ph is associated with two important chromatin functions in spermatocytes—(i) TH2BS11ph is associated with DNA repair domains in the XY body as evidenced by association of TH2BS11ph with repair proteins and histone marks of the XY body (Fig. 7c), (ii) TH2BS11ph could function with H3K.4me3-mediated recruitment of effector proteins and chromatin modellers in regulation of transcription at TSS (Fig. 7d). We would like to point out that the mass spectrometric analysis of proteins associated with TH2BS11ph-containing mononucleosomes and co-existence of H3K4me3, γH2AX by IP assays reflects the average picture of the protein complexes associated with all the TH2BS11ph-containing chromatin domains. Loci-specific recruitment of particular effector molecules, and therefore reprogramming could occur to fine-tune chromatin structure and function at those loci. TH2BS11ph could, therefore, play an important role in creation of chromatin environment along with other histone mark signatures to create platform for XY body and transcription-related functions.

## Conclusions

By immunofluorescence assays with the highly specific antibodies, we demonstrate that TH2BS11ph histone mark is densely localised in the unsynapsed axes of the XY body in pachytene spermatocytes. Genome-wide occupancy of TH2BS11ph histone mark as determined by ChIP sequencing assays further confirmed the localisation of this modification in the X and Y chromosomes. Apart from this, TH2BS11ph is also associated with H3K4me3-containing TSS-associated chromatin domains in P20 (pachytene) and P12 (leptotene) mouse testicular cells. Mass spectrometric analysis of proteins revealed various key proteins linked to functions of the XY body and transcription associated with TH2BS11ph-containing mononucleosomes.

## Materials and methods

### Materials

All fine chemicals were obtained from Sigma Chemicals (USA) unless mentioned otherwise. Synthetic peptides were outsourced from Stork Bio Laboratories (Estonia). The secondary antibodies Donkey anti-mouse, Goat anti-rabbit, Goat anti-rabbit conjugated to Alexa dyes were obtained from Invitrogen (USA). Male Wistar rats and C57BL6 mice were obtained from the Animal Facility, JNCASR. All procedures for handling animals have been approved by the animal ethics committee of the Centre.

### Generation of TH2B-containing nucleosome model

The nucleosome models have been taken from the previous work of our lab by Pentakota et al. [16].

### Purification of in vivo TH2B protein

Histones were isolated from 30- to 35-day-old rat testes by acid extraction method. In vivo TH2B were purified by RP-HPLC technique using the published protocol [16].

### Mass spectrometry for identification of posttranslational modifications on TH2B

#### In-gel digestion

Gel bands were cut into one-mm^3^ pieces and washed twice with MilliQ water. The gel was destained using 1:1 methanol: 50-mM ammonium bicarbonate for 1 min, twice. The gel pieces were dehydrated for 5 min using 1:1 acetonitrile: 50-mM ammonium bicarbonate followed by acetonitrile for 30 s. The gel pieces were dried in a speed-vac (Thermo Savant) for 10 min. The gel pieces were rehydrated in 25-mM dithiothreitol, 50-mM ammonium bicarbonate and incubated at 56 °C for 20 min. After discarding the supernatant, the gel pieces were incubated in 55-mM iodoacetamide at RT for 20 min in the dark and subsequently were washed twice with water, dehydrated and dried as before. The dried gel pieces were rehydrated in 50-mM ammonium bicarbonate containing 250 ng of mass spectrometry grade trypsin (Promega) and incubated overnight at 37 °C. Following digestion, the reaction mixture was acidified with 1% acetic acid and dried in a speed-vac to reduce the volume to 5 µl, to which 10 µl of mobile phase A was added for direct loading for LC–MS/MS analysis.

#### Liquid chromatography–tandem mass spectrometry

Each reaction mixture was analysed by LC–MS/MS. LC was performed on a Easy nanoLC II HPLC system (Thermo Fisher Scientific). Mobile phase A was 94.5% MilliQ water, 5% acetonitrile, 0.5% acetic acid. Mobile phase B was 80% acetonitrile, 19.5% MilliQ water, 0.5% acetic acid. The 120-min LC gradient ran from 2% B to 35% B over 90 min, with the remaining time used for sample loading and column regeneration. Samples were loaded to a 2 cm × 100 μm I.D. trap column positioned on an actuated valve (Rheodyne). The column was 13 cm × 100 μm I.D. fused silica with a pulled tip emitter. Both trap and analytical columns were packed with 3.5 µm C18 resin (Zorbax SB, Agilent). The LC was interfaced to a dual pressure linear ion-trap mass spectrometer (LTQ Velos, Thermo Fisher) via nano-electrospray ionisation. An electrospray voltage of 1.8 kV was applied to a pre-column tee. The mass spectrometer was programmed to acquire, by data-dependent acquisition, tandem mass spectra from the top 15 ions in the full scan from 400 to 1400 m/z. Dynamic exclusion was set to 30 s.

#### Data processing and library searching

Mass spectrometer RAW data files were converted into MGF format using msconvert. Detailed search parameters are printed in the search output XML files. Briefly, all searches required strict tryptic cleavage, 0 or 1 missed cleavages, fixed modification of cysteine alkylation, variable modification of methionine oxidation and expectation value scores of 0.01 or lower. MGF files were searched using X! Hunter against the latest library available on the GPM at the time. Other searches used the cRAP contaminant library from the GPM and libraries constructed from the most recent ENSEMBL release available at the time. MGF files were searched using X!! Tandem using both the native and k-score5 scoring algorithms and by OMSSA. All searches were performed on Amazon Web Services-based cluster compute instances using the Proteome Cluster interface. XML output files were parsed, and non-redundant protein sets determined using in-house scripts. Proteins were required to have 2 or unique peptides with *E*-value scores of 0.01 or less, 0.001 for X!Hunter and protein *E*-value scores of 0.0001 or less.

### Alignment of the amino acid sequences

Multiple sequence alignment (MSA) was performed for TH2B of selected mammals to elucidate the sequence conservation across species.

### Antibody generation

Peptides corresponding to TH2BS11ph modification (CKGTTI(pS)KKGFK), H2B(KSRPAPKKGSK) were injected into rabbits, and the 14-day cycle of antibody generation was followed. Immunoglobulins were purified by caprylic acid based purification. Peptide-affinity-based purification with the Sulfolink columns containing immobilised peptides was used to purify the TH2BS11ph- and H2B-specific antibodies. The TH2BS11ph antibody was outsourced from Abgenex company.

### Preparation of nuclear lysates

Nuclear lysates were prepared by the method described previously with modifications [72]. Briefly, testes were dissected in cytoplasmic lysis buffer (10-mM HEPES pH 7.5, 50-mM NaCl, 0.5-M sucrose, 0.5% Triton-X-100, 0.1-mM EDTA, 1-mM DTT, protease inhibitor cocktail), incubated on ice for 15 min and centrifuged at 1500*g* for 7 min. The nuclear pellet was resuspended in Buffer B1 (10-mM HEPES pH 7.5, 500-mM NaCl, 0.1-mM EDTA, 1-mM DTT, 0.5% NP-40, protease inhibitor cocktail) to obtain nuclear lysates or Buffer B2 (10-mM HEPES, 200-mM NaCl, 1-mM EDTA, 0.5% NP-40, protease inhibitor cocktail) for isolation of chromatin. The nuclear lysates were clarified by centrifugation at 15,100×*g* for 10 min.

### ELISA

Peptides were used at 200 ng per well. The pre-bleed and immune sera were used at 1:5000 dilution. Goat anti-rabbit HRP were used as the secondary antibody at 1:5000 dilution. TMB (3,3′,5,5′-tetramethylbenzidine) was used as the substrate for the reaction. After 3 min of enzyme–substrate reaction, the plate was read at 450 nm.

### Preparation of meiotic spreads

Meiotic spreads were prepared using the published protocol [73]. Briefly, testes were decapsulated and chopped in PBS solution (pH 7.4). The cell pellet was resuspended in hypotonic buffer (30-mM Tris, 17-mM sodium citrate, 50-mM sucrose, 5-mM EDTA, 0.5-mM DTT, protease inhibitor cocktail) and incubated for 30 min. The pellet was resuspended in 100-mM sucrose solution, and the nuclei were spread onto PFA-coated slides. The slides were kept for drying at room temperature for 2 h and proceeded for immunofluorescence studies.

### Immunofluorescence

The slides were kept in blocking solution (3% BSA solution in PBS) for 1 h at room temperature; then treated with primary antibody overnight in the cold room, washed with 0.1% PBST solution and then incubated with secondary antibody for 1 h at room temperature. Next, washes were given with PBST solution and the smears were mounted using DAPI solution. Images were acquired by Zeiss confocal laser scanning microscope (LSM880 or LSM510). Zen software was used for image analysis. Pearson Correlation Coefficient was computed to determine the overlap between the two channels. To evaluate specific colocalization, using ImageJ (Fiji) software, we rotated the red channel in the images by 90° in the anticlockwise direction in the XY plane. Pearson Correlation Coefficient was computed to evaluate colocalization percentages upon rotation of images captured in the red channel [74, 75]. Colocalization percentages were calculated multiplying the Pearson Correlation Coefficient by 100. All data were confirmed with at least three independent mice and rats.

### Isolation of mononucleosomes

Immunoprecipitation using mononucleosomes was carried out as described [12]. Briefly, mouse testes were dissected and homogenised in lysis buffer (60-mM KCl, 15-mM NaCl, 15-mM Tris–HCl, 0.03% Triton-X-100, 0.34-M Sucrose, 2-mM EDTA, 0.5-mM EGTA, 0.65-mM spermidine, 1-mM DTT, 1% glycerol, protease and phosphatase inhibitor cocktail), centrifuged at 650×*g* for 10 min at 4 °C. The pellet was washed with wash buffer containing 60-mM KCl, 15-mM NaCl, 15-mM Tris–HCl, 0.34-M Sucrose, 0.5-mM EGTA, 1-mM DTT, 0.5-mM PMSF and protease and phosphatase inhibitor cocktail. The pellet was resuspended in MNase buffer (10-mM Tris–HCl, 10-mM KCl, and 2-mM CaCl_2_). The nucleosome fraction was isolated by centrifugation at 650×*g* for 10 min at 4 °C, mixed with LSDB250 buffer (20% glycerol, 50-mM HEPES, 3-mM MgCl_2_, 250-mM KCl, protease and phosphatase inhibitor cocktail) and proceeded with the immunoprecipitation protocol.

### ChIP sequencing of TH2BS11ph modification-associated chromatin in P20 and P12 mouse testicular cells

DNA was isolated from TH2BS11ph immunoprecipitated mononucleosomes by phenol–chloroform method. The quality control of the DNA samples was done using the Qubit and Tapestation methods. The libraries were subjected for 40 million depth paired-end (100 bp × 2) sequencing that was carried using Illumina HiSeq 2500. FASTQ files were obtained and data analyses were carried out further.

### Data analysis

FASTQ files were aligned to mm10 genome assembly using Bowtie2 [76]. While aligning, unpaired and discordant reads were removed. The aligned files were sorted and indexed accordingly, and also made free from PCR duplicates. On average for all samples, read-alignment rate appeared to be higher than 70%. Principal Component Analysis (PCA) was performed to evaluate the correlation between the aligned samples of each condition. The sorted aligned replicates of background TH2B and antibody-treated (IP) TH2BS11ph were merged, respectively, using Samtools Merge [70]. Unique peaks were obtained by performing the peak calling of TH2BS11ph data against the published backbone TH2B ChIP seq data. The peaks were called between the control and IP files using SICER 1.1 Version [77]  with the following parameters—Redundancy threshold: 1; Window size: 200 bp; Fragment size: 150 bp; Gap size: 600 bp; FDR: 0.01. The final peaks were shortlisted giving the cutoff of > 1.5 fold change.

### Aggregation plot (NGS plot)

ngs.plot.r [78] was used to plot read count per million mapped reads for each ChIP samples (P20 TH2BS11ph, background TH2B, P12 TH2BS11ph)individually against the genome-wide coordinates of the following datasets—total H3K4me3, common H3K4me3, transcription-specific H3K4me3, TSS, and Total hotspots. Furthermore, ngs.plot.r was also used to plot log2(Fold change vs. Control) of in-house ChIP-seq sample (P20 TH2BS11ph) over the background TH2B dataset for each of the following genome-wide coordinates of Total H3K4me3, common H3K4me3, transcription-specific H3K4me3 (H3K4me3 common), TSS, and Total DSB hotspots.

### Primer design for ChIP-PCR studies

Peak summits corresponding to high TH2BS11ph occupancy were chosen for experimental validation using ChIP-PCR. To maintain the rigour of primer design, primers were designed using the Primer BLAST and primer 3 tools and were also verified computationally using NCBI Primer Blast and UCSC In-silico PCR. Verification of primer-dimer formation was also considered during the design (Additional file 6: Table S1).

### Immunoprecipitation and quantitative PCR

The mononucleosome fraction was incubated with either anti-γH2AX (Upsate, 05-636) or anti-H3K4me3 (Abcam, ab12209) or anti-TH2BS11ph for overnight at 4 °C. Protein A or Protein G dynabeads were added the next day. LSDB250 buffer was used as the wash buffer for immunoprecipitation studies with mononucleosomes. After the washes, the beads were either proceeded with DNA extraction for PCR analysis or boiled in 5× SDS dye for western blotting. After washing of beads, DNA was eluted from the beads as follows—210 µl of the elution buffer was added, incubated at 65 °C overnight for de-crosslinking. 200 µl of TE buffer was added the next day, subjected for RNase (final; 40 µg/ml) and proteinase K (Final; 100 µg/ml) treatment and DNA were extracted by phenol–chloroform method. DNA that was purified from TH2BS11ph ChIP was proceeded for ChIP-seq analyses. SYBR kit from TAKARA was used to set up quantitative PCR reactions. PCR was carried out for 40 cycles and was followed by melt curve analyses before recording the raw Ct values. The fold enrichment values were calculated over input taking the percentage of input used for the ChIP procedure and the Ct values obtained for the target genomic region from Input and ChIP DNA. PCR was carried out in duplicates for each of the three biological replicates.

$${\text{Fold}}\;\;{\text{Enrichment}}\;\;{\text{over}}\;\;{\text{Input}}\; = \% \;{\text{of}}\;{\text{Input}}\; \times 2^{{\left[ {{\text{Ct}}\left( {\text{Input}} \right) - {\text{Ct}}\left( {\text{ChIP}} \right)} \right]}}$$

### Mass spectrometric identification of interacting protein partners of TH2BS11ph-containing mononucleosomes

Immunoprecipitation of TH2BS11ph-containing proteins were carried out and the proteins were extracted from the beads using the elution buffer of the Pierce co-IP kit. The eluted proteins were resolved on 15% SDS gel and the gel was subjected to Coomassie staining. The stained wells corresponding to the IgG and IP lanes were outsourced for mass spectrometry to identify the interacting proteins.

#### Methods for protein sequence analysis by LC–MS/MS

Excised gel bands were cut into approximately 1-mm^3^ pieces. Gel pieces were then subjected to a modified in-gel trypsin digestion procedure [79]. Gel pieces were washed and dehydrated with acetonitrile for 10 min. followed by removal of acetonitrile. Pieces were then completely dried in a speed-vac. Rehydration of the gel pieces was with 50-mM ammonium bicarbonate solution containing 12.5 ng/µl modified sequencing-grade trypsin (Promega, Madison, WI) at 4 °C. After 45 min., the excess trypsin solution was removed and replaced with 50-mM ammonium bicarbonate solution to just cover the gel pieces. Samples were then placed in a 37 °C room overnight. Peptides were later extracted by removing the ammonium bicarbonate solution, followed by one wash with a solution containing 50% acetonitrile and 1% formic acid. The extracts were then dried in a speed-vac (~ 1 h). The samples were then stored at 4 °C until analysis.

On the day of analysis the samples were reconstituted in 5–10 µl of HPLC solvent A (2.5% acetonitrile, 0.1% formic acid). A nano-scale reverse-phase HPLC capillary column was created by packing 2.6 µm C18 spherical silica beads into a fused silica capillary (100 µm inner diameter × ~ 30 cm length) with a flame-drawn tip [80]. After equilibrating the column, each sample was loaded via a Famos auto sampler (LC Packings, San Francisco CA) onto the column. A gradient was formed and peptides were eluted with increasing concentrations of solvent B (97.5% acetonitrile, 0.1% formic acid).

As peptides eluted, they were subjected to electrospray ionisation and then entered into an LTQ Orbitrap Velos Pro ion-trap mass spectrometer (Thermo Fisher Scientific, Waltham, MA). Peptides were detected, isolated, and fragmented to produce a tandem mass spectrum of specific fragment ions for each peptide. Peptide sequences (and hence protein identity) were determined by matching protein databases with the acquired fragmentation pattern by the software programme, Sequest (Thermo Fisher Scientific, Waltham, MA) [81]. All databases include a reversed version of all the sequences and the data was filtered to between a one and two percent peptide false discovery rate along with filter to being set to at least 1 unique peptide per protein (Additional file 7: Table S2).

### Dot-blot analysis

Two µg of peptides corresponding to TH2B (CKGTTISKKGFK), TH2BS11ph (CKGTTI(pS)KKGFK),H2B(KSRPAPKKGSK),H2BS14ph(SRPAPKKG(pS)KKC) was applied as separate spots on the nitrocellulose membrane. After drying, the blot was subjected to steps of western blotting with the TH2BS11ph antibody.

### Western blot analysis

For western blot, proteins were resolved by SDS-PAGE gel electrophoresis and then transferred onto a nitrocellulose membrane using the semi-dry transfer technique. The membrane was blocked using 5% skimmed milk or 3% BSA (diluted in TBS) for 1 h at room temperature, then incubated with the specific primary antibody for 1 h at room temperature or overnight at 4 °C. The blots were given multiple washes with 0.1% PBST or TBST for 10 min each. Next, the blot was incubated with the secondary antibody (anti-rabbit/anti-mouse) for 1 h at room temperature. Membranes were washed extensively with 0.1% PBST or TBST and developed using the ECL kit (Thermo Scientific). For the peptide competition assay, 50-fold molar excess of the modified and unmodified peptides were added to the antibody solution and mixed for 3 h at 4 °C before the addition to the blot.

## Supplementary information

**Additional file 1: Figure S1.** Validation of H2B-specific antibody. H2B antibody was successfully generated in rabbits and the staining pattern was found to not coincide with the XY body in the pachytene spermatocyte. **A** Fragmentation table for the PTM serine 11 phosphorylation obtained on TH2B. **B** Dot-blot assay demonstrating the specificity of TH2BS11ph antibody wherein we show the specificity of the antibody towards the serine 11 phosphopeptide but does not cross-react with backbone TH2B, backbone H2B or H2B Serine 14 phospho peptide. **C** Specificity of the commercial H2BS14ph antibody as shown by immunoblotting against liver histones, testis histones and HPLC-purified in vivo TH2B (labelled ‘in vivo TH2B’). This antibody cross-reacts with TH2B as can be seen by its reactivity towards testis nuclear lysates and in vivo TH2B. **D** Validation of H2B antibody by dot-blot [first panel]; the first lane represents reactivity of the H2B with the non-phosphorylated H2B peptide, second lane represents reactivity with the serine 14 phosphorylated H2B peptide. Immunoblotting of H2B antibody against liver histones, testis histones and in vivo TH2B [Second panel]. **E** Immunostaining of anti-H2B and anti-Scp3 antibodies across the three stages of meiotic prophase I-leptotene, zygotene and pachytene intervals. Nuclei were visualised by DAPI staining, Scale bars, 10 µm.

**Additional file 2: Figure S2.** Colocalization studies of TH2BS11ph with Scp3, γH2AX, Rad51, pATM and Spo11 in rat pachytene spermatocytes. TH2BS11ph colocalizes with Scp3, γH2AX, Spo11, Rad51 and pATM in rat pachytene spermatocytes, a clear colocalization seen in axes of the XY body. **A** Colocalization studies of TH2BS11ph with Scp3 across leptotene (1st panel), zygotene (2nd panel) and pachytene (3rd panel) intervals in meiotic spreads in rats. **B** Colocalization studies of TH2BS11ph with γH2AX in rat spermatocytes in pachytene spermatocytes in rat meiotic spreads. **C** Colocalization studies of TH2BS11ph with Rad51 in pachytene stage of rat spermatocytes. **D** Immunofluorescence studies of TH2BS11ph with pATM in pachytene spermatocyte of rat. **E** Immunofluorescence studies of TH2BS11ph with Spo11 in pachytene spermatocyte of rat. The inset in all the figures shows the XY body in all the pachytene cells. All data were confirmed with at least three independent rats. Nuclei were visualised by DAPI staining, Scale bars, 10 µm.

**Additional file 3: Figure S3.** Read distribution of TH2BS11ph histone mark at TSS and recombination hotspots in mouse P20 testicular cells. Read Profile of TH2BS11ph at TSS and recombination hotspots in P20 testicular cells. Read distribution of TH2BS11ph at **A** Centre of total H3K4me3 marks; **B** DSB hotspots; **C** TSS-associated H3K4me3, **D** Total TSS of mouse obtained from UCSC; **E** Chromosome X-specific H3K4me3;. The read distribution was plotted in terms of aggregation plots (first panels in Fig (**A**–**D**) and heat maps (second panels in Fig (**A**–**D**). *X*-axis in all the aggregation plots represents read count per million mapped reads whereas *Y*-axis represents the distance from the centre of the reference peak in kilobase pairs (kb).

**Additional file 4: Figure S4.** Read distribution of TH2BS11ph histone mark at TSS and recombination hotspots in mouse P12 testicular cells. Genome-wide occupancy of TH2BS11ph modification in P12 testicular cells. **A** Aggregation plot and Heat map for analysis of overlap of TH2BS11ph with Total H3K4me3; **B** Aggregation plot and Heat map for determining localisation of TH2BS11ph at DSB hotspots; **C** Aggregation plot and Heat map for analysis of overlap of TH2BS11ph at H3K4me3 associated TSS, **D** Aggregation plot and Heat map for analysis of association of TH2BS11ph at Transcription Start Sites (TSS) of mouse. *X*-axis in all the aggregation plots represents read count per million mapped reads whereas *Y*-axis represents the distance from the centre of the reference peak in kilobase pairs (kb).

**Additional file 5: Figure S5.** Pattern of digestion of DNA fragments obtained after MNase digestion of chromatin of mouse testicular cells. **A** Profile of chromatin fragments obtained after MNase digestion for various time points in mouse testes; **B** Specificity of TH2BS11ph antibody in the immunoprecipitation reaction—the first lane refers to input fraction, the second lane refers to IP with non-specific rabbit IgG; the third lane refers to the TH2BS11ph containing ChIP fraction whereas the fourth lane refers to TH2BS11ph IP carried out along with the addition of competing TH2B serine 11 phosphopeptide.

**Additional file 6: Table S1.** Table of primer sequences.

**Additional file 7: Table S2.** Table of Mass spectrometry dataset.

**Additional file 8: Table S2.** ChIP-seq dataset [P20]. TH2BS11ph ChIP sequencing peaks (called over TH2B dataset) in P20 mouse testicular cells.

**Additional file 9: Table S3.** ChIP-seq dataset [P12]. TH2BS11ph ChIP sequencing peaks (called over TH2B dataset) in P12 mouse testicular cells.

## Data Availability

The ChIP-sequencing dataset containing the raw and processed files have been deposited in the Gene Expression Omnibus (Accession Number—GSE135209) (Additional file 8: Table S3 and Additional file 9: Table S4).

## Abbreviations

DDR: DNA damage response
ATM: ataxia-telangiectasia-mutated
ATR: ataxia telangiectasia and Rad3-related
PRDM9: PR domain-containing protein 9
Smarca5: SWI/SNF-related, matrix-associated, actin-dependent regulator of chromatin, subfamily A, member 5
Scml2: sex comb on midleg-like protein 2
Mre11: meiotic recombination 11
Cbx1: chromobox protein homolog 1
HORMAD1: HORMA (Hop1, Rev7 and Mad2) domain-containing protein 1
Brdt: bromodomain, testis-specific
