# Supplementary figures and images for: TH2BS11ph histone mark is enriched in the unsynapsed axes of the XY body and predominantly associates with H3K4me3-containing genomic regions in mammalian spermatocytes
Source: Epigenetics Chromatin. 2019 Sep 7;12:53. doi: 10.1186/s13072-019-0300-y (PMC6731575; doi:10.1186/s13072-019-0300-y)

Figure S1

A)

| bond | +1y      | +1y-17   | +1y-18   | +1b      | +1b-17   | +1b-18   | +2y     | +2y-17  | +2y-18  | +2b     | +2b-17  | +2b-18  |
|------|----------|----------|----------|----------|----------|----------|---------|---------|---------|---------|---------|---------|
| P1   | 1532.772 | 1515.746 | 1514.762 | 98.060   | 81.034   | 80.050   | 766.890 | 758.376 | 757.884 | 49.534  | 41.020  | 40.528  |
| E2   | 1403.730 | 1386.703 | 1385.719 | 227.103  | 210.076  | 209.092  | 702.368 | 693.855 | 693.363 | 114.055 | 105.542 | 105.050 |
| V3   | 1304.661 | 1287.635 | 1286.651 | 326.171  | 309.145  | 308.161  | 652.834 | 644.321 | 643.829 | 163.589 | 155.076 | 154.584 |
| S4   | 1217.629 | 1200.603 | 1199.619 | 413.203  | 396.177  | 395.193  | 609.318 | 600.805 | 600.313 | 207.105 | 198.592 | 198.100 |
| A5   | 1146.592 | 1129.566 | 1128.582 | 484.240  | 467.214  | 466.230  | 573.800 | 565.286 | 564.794 | 242.624 | 234.111 | 233.619 |
| K6   | 1018.497 | 1001.471 | 1000.487 | 612.335  | 595.309  | 594.325  | 509.752 | 501.239 | 500.747 | 306.671 | 298.158 | 297.666 |
| Q7   | 961.476  | 944.449  | 943.465  | 669.357  | 652.330  | 651.346  | 481.241 | 472.728 | 472.236 | 335.182 | 326.669 | 326.177 |
| T8   | 860.428  | 843.401  | 842.417  | 770.404  | 753.378  | 752.394  | 430.718 | 422.204 | 421.712 | 385.706 | 377.193 | 376.701 |
| T9   | 759.380  | 742.354  | 741.370  | 871.452  | 854.426  | 853.442  | 380.194 | 371.681 | 371.189 | 436.230 | 427.716 | 427.224 |
| I10  | 646.296  | 629.270  | 628.286  | 984.536  | 967.510  | 966.526  | 323.652 | 315.138 | 314.646 | 492.772 | 484.258 | 483.766 |
| S11  | 479.298  | 462.271  | 461.287  | 1151.534 | 1134.508 | 1133.524 | 240.153 | 231.639 | 231.147 | 576.271 | 567.758 | 567.266 |
| K12  | 351.203  | 334.176  | 333.192  | 1279.629 | 1262.603 | 1261.619 | 176.105 | 167.592 | 167.100 | 640.318 | 631.805 | 631.313 |
| K13  | 223.108  | 206.081  | 205.097  | 1407.724 | 1390.698 | 1389.714 | 112.058 | 103.544 | 103.052 | 704.366 | 695.853 | 695.361 |
| Q14  | 166.087  | 149.060  | 148.076  | 1464.746 | 1447.719 | 1446.735 | 83.547  | 75.034  | 74.542  | 732.877 | 724.363 | 723.871 |

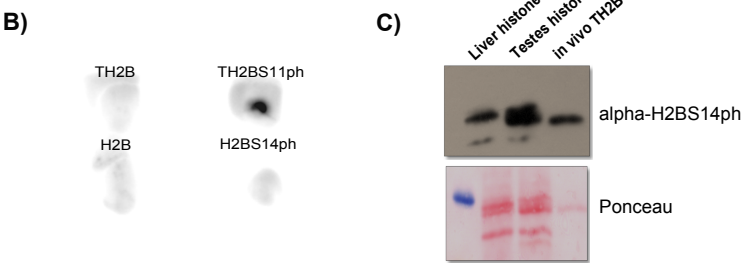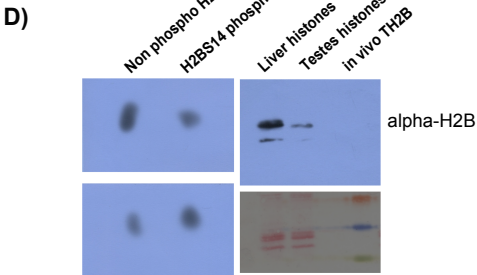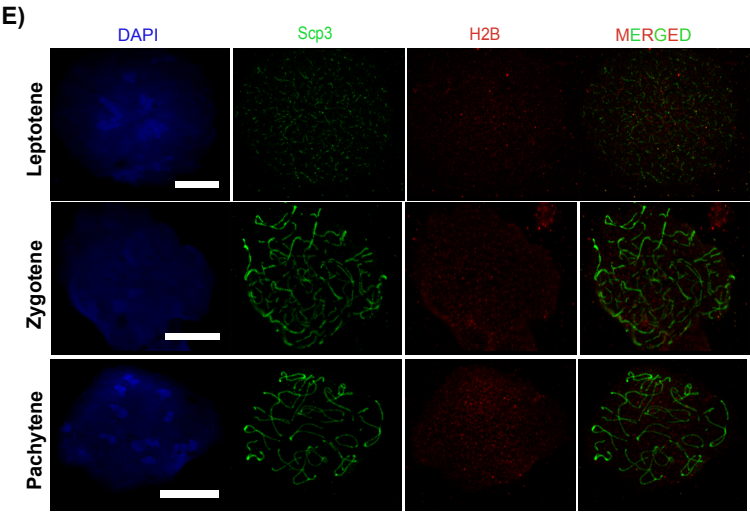

Supplement: Supplementary file 1 — Additional file 1: Figure S1. Validation of H2B-specific antibody. H2B antibody was successfully generated in rabbits and the staining pattern was found to not coincide with the XY body in the pachytene spermatocyte. A Fragmentation table for the PTM serine 11 phosphorylation obtained on TH2B. B Dot-blot assay demonstrating the specificity of TH2BS11ph antibody wherein we show the specificity of the antibody towards the serine 11 phosphopeptide but does not cross-react with backbone TH2B, backbone H2B or H2B Serine 14 phospho peptide. C Specificity of the commercial H2BS14ph antibody as shown by immunoblotting against liver histones, testis histones and HPLC-purified in vivo TH2B (labelled ‘in vivo TH2B’). This antibody cross-reacts with TH2B as can be seen by its reactivity towards testis nuclear lysates and in vivo TH2B. D Validation of H2B antibody by dot-blot [first panel]; the first lane represents reactivity of the H2B with the non-phosphorylated H2B peptide, second lane represents reactivity with the serine 14 phosphorylated H2B peptide. Immunoblotting of H2B antibody against liver histones, testis histones and in vivo TH2B [Second panel]. E Immunostaining of anti-H2B and anti-Scp3 antibodies across the three stages of meiotic prophase I-leptotene, zygotene and pachytene intervals. Nuclei were visualised by DAPI staining, Scale bars, 10 µm. [file 13072_2019_300_MOESM1_ESM.pdf]

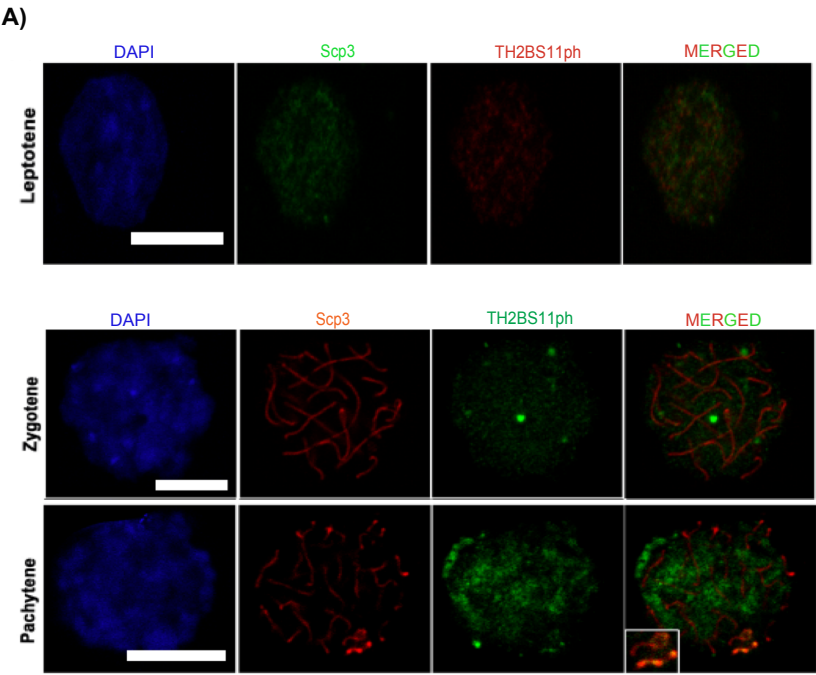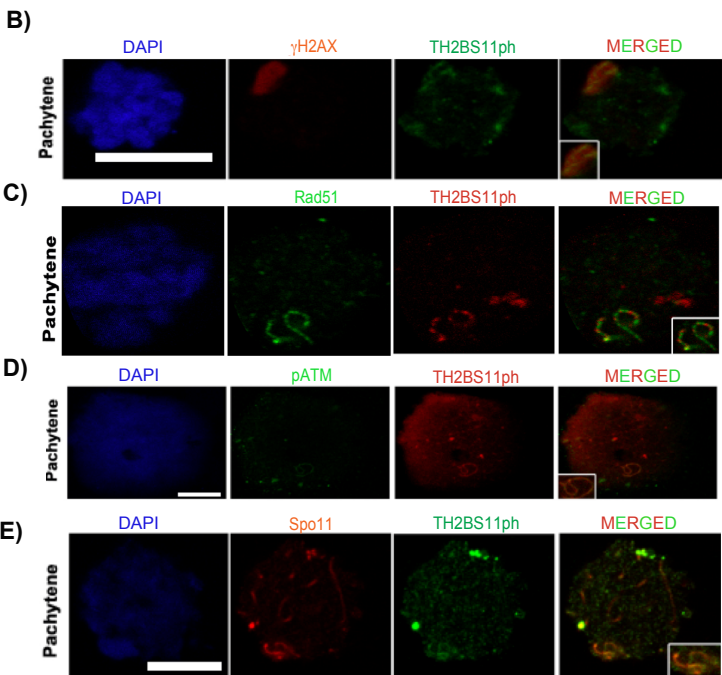

Supplement: Supplementary file 2 — Additional file 2: Figure S2. Colocalization studies of TH2BS11ph with Scp3, γH2AX, Rad51, pATM and Spo11 in rat pachytene spermatocytes. TH2BS11ph colocalizes with Scp3, γH2AX, Spo11, Rad51 and pATM in rat pachytene spermatocytes, a clear colocalization seen in axes of the XY body. A Colocalization studies of TH2BS11ph with Scp3 across leptotene (1st panel), zygotene (2nd panel) and pachytene (3rd panel) intervals in meiotic spreads in rats. B Colocalization studies of TH2BS11ph with γH2AX in rat spermatocytes in pachytene spermatocytes in rat meiotic spreads. C Colocalization studies of TH2BS11ph with Rad51 in pachytene stage of rat spermatocytes. D Immunofluorescence studies of TH2BS11ph with pATM in pachytene spermatocyte of rat. E Immunofluorescence studies of TH2BS11ph with Spo11 in pachytene spermatocyte of rat. The inset in all the figures shows the XY body in all the pachytene cells. All data were confirmed with at least three independent rats. Nuclei were visualised by DAPI staining, Scale bars, 10 µm. [file 13072_2019_300_MOESM2_ESM.pdf]

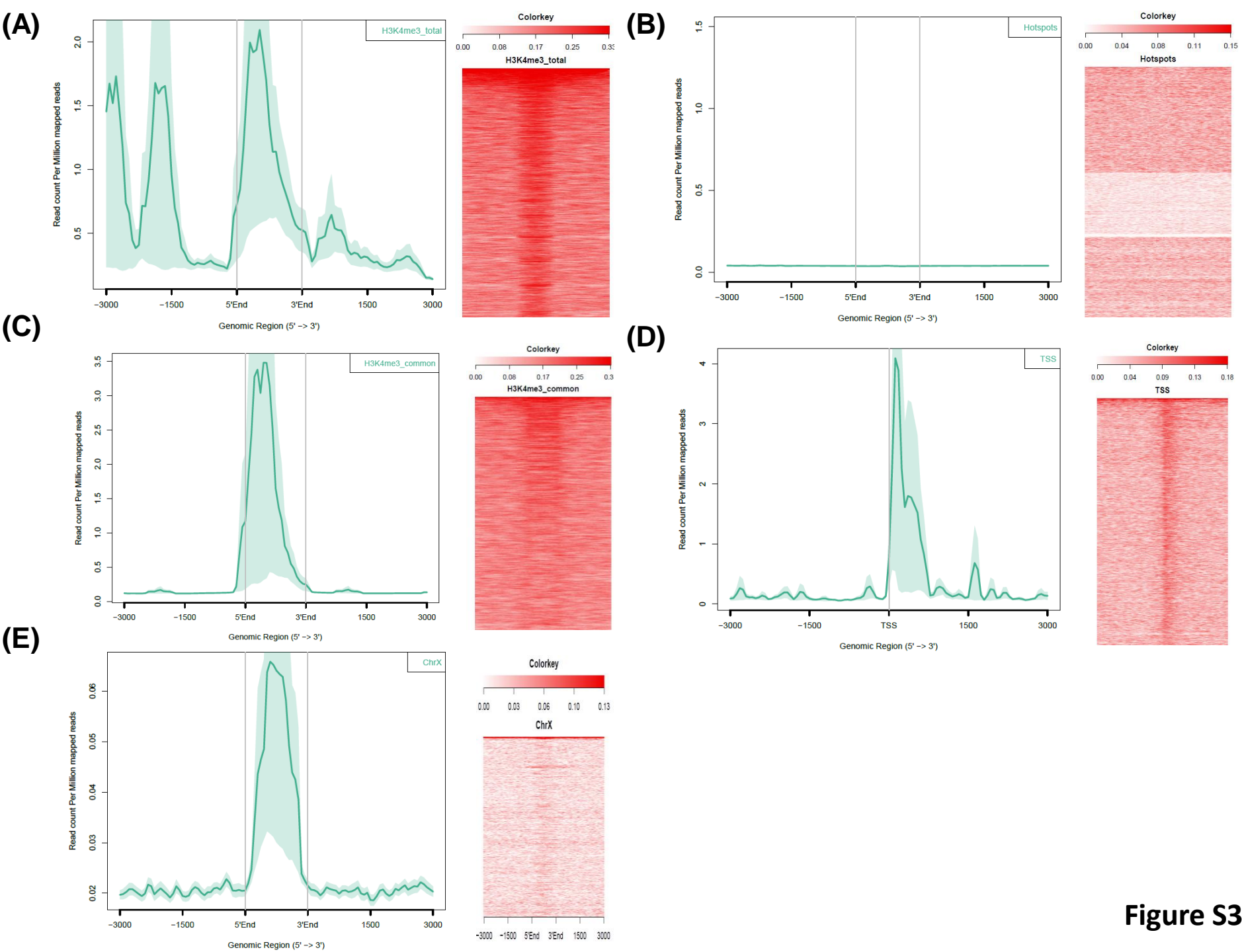

**Figure S3**

Supplement: Supplementary file 3 — Additional file 3: Figure S3. Read distribution of TH2BS11ph histone mark at TSS and recombination hotspots in mouse P20 testicular cells. Read Profile of TH2BS11ph at TSS and recombination hotspots in P20 testicular cells. Read distribution of TH2BS11ph at A Centre of total H3K4me3 marks; B DSB hotspots; C TSS-associated H3K4me3, D Total TSS of mouse obtained from UCSC; E Chromosome X-specific H3K4me3;. The read distribution was plotted in terms of aggregation plots (first panels in Fig (A–D) and heat maps (second panels in Fig (A–D). X-axis in all the aggregation plots represents read count per million mapped reads whereas Y-axis represents the distance from the centre of the reference peak in kilobase pairs (kb). [file 13072_2019_300_MOESM3_ESM.pdf]

**Figure S4**

**(A)**

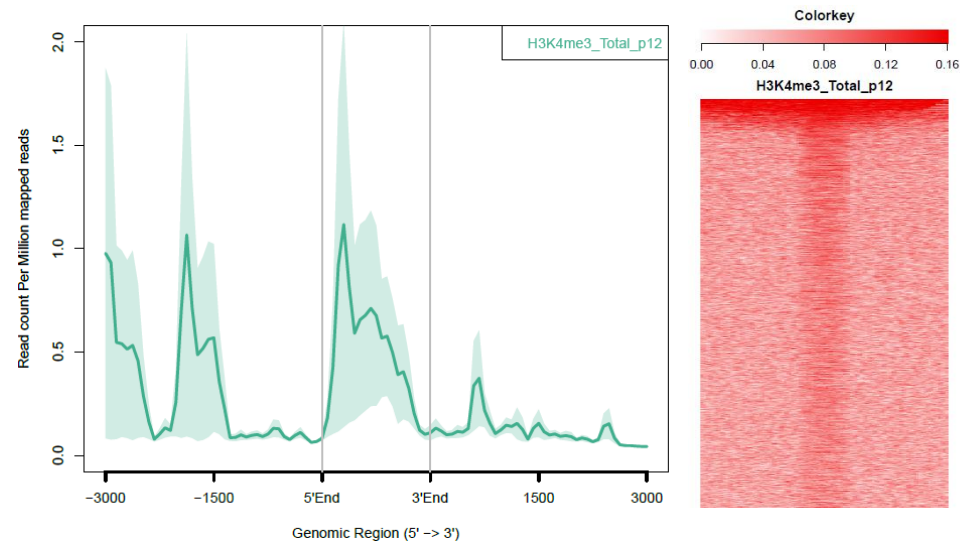

**(B)**

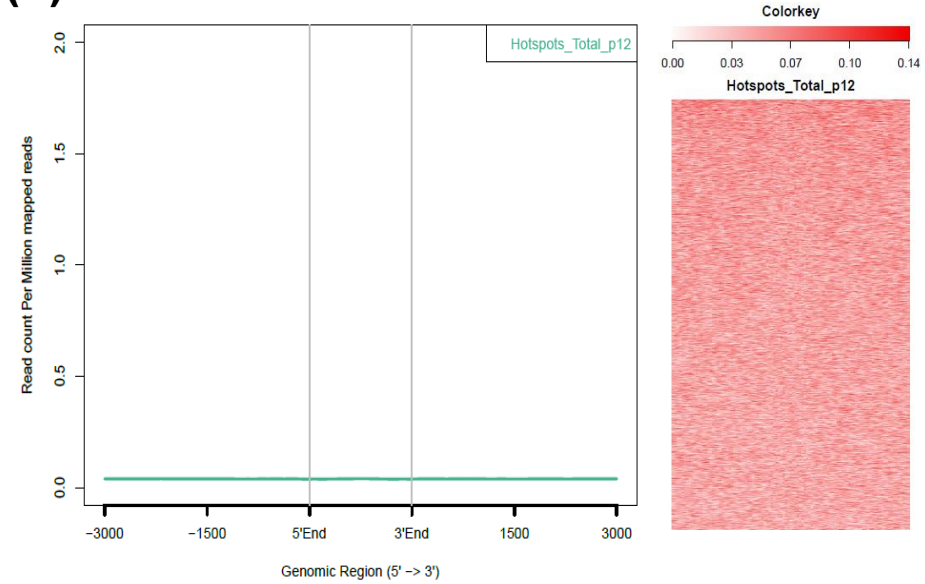

**(C)**

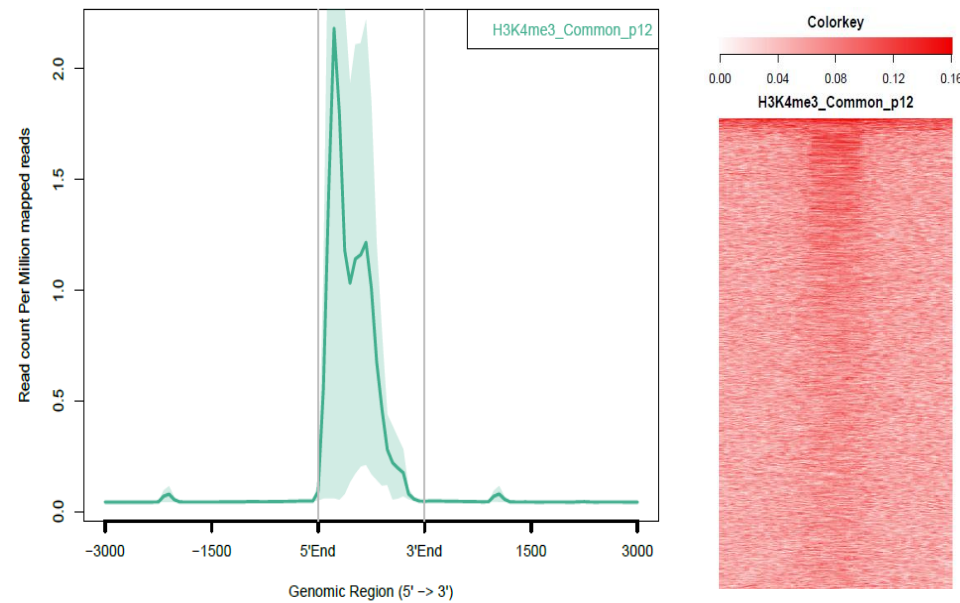

**(D)**

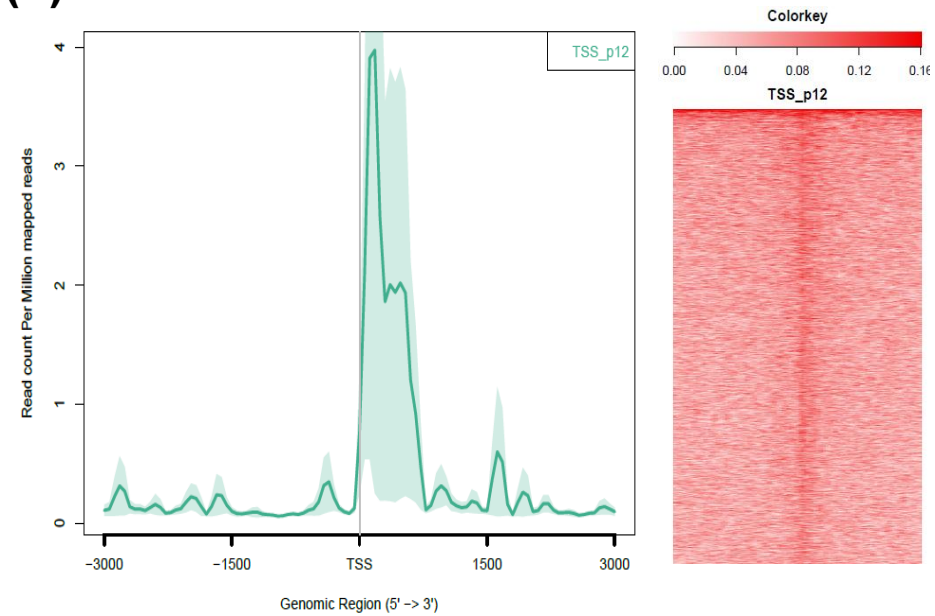

Supplement: Supplementary file 4 — Additional file 4: Figure S4. Read distribution of TH2BS11ph histone mark at TSS and recombination hotspots in mouse P12 testicular cells. Genome-wide occupancy of TH2BS11ph modification in P12 testicular cells. A Aggregation plot and Heat map for analysis of overlap of TH2BS11ph with Total H3K4me3; B Aggregation plot and Heat map for determining localisation of TH2BS11ph at DSB hotspots; C Aggregation plot and Heat map for analysis of overlap of TH2BS11ph at H3K4me3 associated TSS, D Aggregation plot and Heat map for analysis of association of TH2BS11ph at Transcription Start Sites (TSS) of mouse. X-axis in all the aggregation plots represents read count per million mapped reads whereas Y-axis represents the distance from the centre of the reference peak in kilobase pairs (kb). [file 13072_2019_300_MOESM4_ESM.pdf]

**Figure S5**

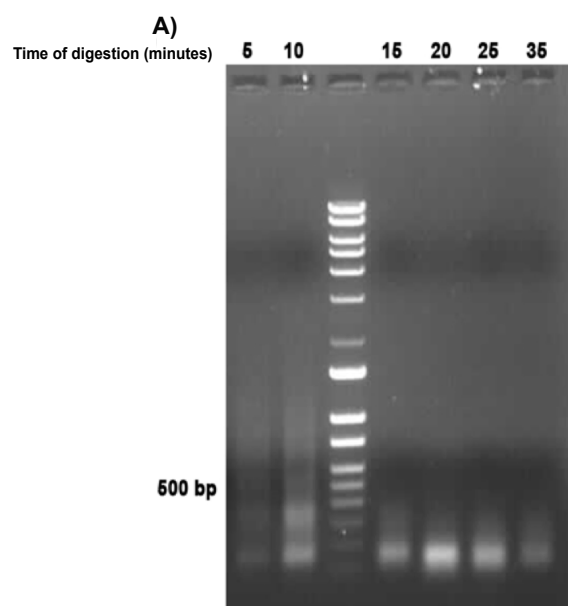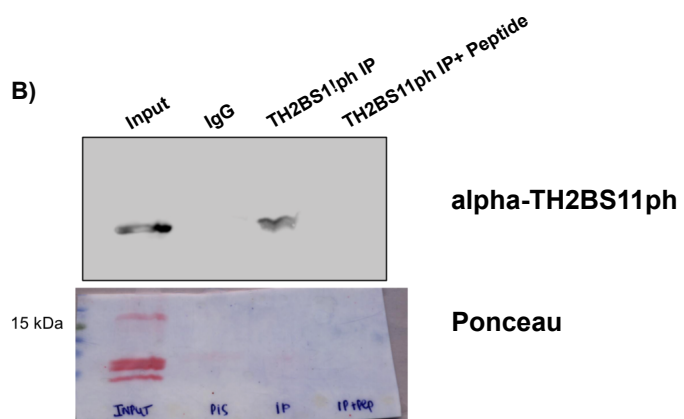

Supplement: Supplementary file 5 — Additional file 5: Figure S5. Pattern of digestion of DNA fragments obtained after MNase digestion of chromatin of mouse testicular cells. A Profile of chromatin fragments obtained after MNase digestion for various time points in mouse testes; B Specificity of TH2BS11ph antibody in the immunoprecipitation reaction—the first lane refers to input fraction, the second lane refers to IP with non-specific rabbit IgG; the third lane refers to the TH2BS11ph containing ChIP fraction whereas the fourth lane refers to TH2BS11ph IP carried out along with the addition of competing TH2B serine 11 phosphopeptide. [file 13072_2019_300_MOESM5_ESM.pdf]
