# Supplementary material for: TH2BS11ph histone mark is enriched in the unsynapsed axes of the XY body and predominantly associates with H3K4me3-containing genomic regions in mammalian spermatocytes
Source: Epigenetics Chromatin. 2019 Sep 7;12:53. doi: 10.1186/s13072-019-0300-y (PMC6731575; doi:10.1186/s13072-019-0300-y)
Supplement: Supplementary file 6 — Additional file 6: Table S1. Table of primer sequences. [file 13072_2019_300_MOESM6_ESM.docx]

**Additional File 6- Table_1- Table of Primer Sequences**

| **Label** | **Sequence 5'-3'** |
| --- | --- |
| chrX1 FP | AAAGAAGAGCTTCCAACTGGTTTAAGT |
| chr X1 RP | CTACAAATTATTTTGACCCTTACCTCA |
| chrX2 FP | AACCTACTCCTACTTTCATGGGTTTTT |
| chrX2 RP | GTAATTAAGCAGTAAGCGACATCGAAT |
| chrY FP | AAAATTTTGAGGGTTTTATGGCTCTTTTAG |
| chrY RP | CCCTAAAACATTAAAGCAAAAACTAATACC |
| Auto1 FP | TCCACCTTGAGTTGTGAGGC |
| Auto1 RP | GGATTTGGGGCCAGGTACTC |
| Auto2 FP | AACCCTAAATCCTTCCCATCACAA |
| Auto2 RP | TCTTCACTTTTGCTCGAAAGACAC |
| Neg Ctrl1 FP | ACTTATCGCCTTTCCTGTAGGG |
| Neg Ctrl1 RP | TTCTGAGGAATATTGCCTGCCC |
| Neg Ctrl2 FP | ACGCTGGGGTGTACAAATCT |
| Neg Ctrl2 RP | TATGCCTTTGAGACGCTCCC |
